# Three new serine-protease autotransporters of *Enterobacteriaceae* (SPATEs) from extra-intestinal pathogenic *Escherichia coli* and combined role of SPATEs for cytotoxicity and colonization of the mouse kidney

**DOI:** 10.1080/21505594.2019.1624102

**Published:** 2019-06-14

**Authors:** Hajer Habouria, Pravil Pokharel, Segolène Maris, Amélie Garénaux, Hicham Bessaiah, Sébastien Houle, Frédéric J. Veyrier, Stéphanie Guyomard-Rabenirina, Antoine Talarmin, Charles M. Dozois

**Affiliations:** a Institut national de recherche scientifique (INRS)-Institut Armand Frappier, Laval, Quebec, Canada; b Centre de recherche en infectiologie porcine et avicole (CRIPA); c Institut Pasteur International Network; d Unité Environnement Santé, Institut Pasteur de Guadeloupe, Les Abymes, Guadeloupe, France

**Keywords:** *Escherichia coli*, Autotransporters, serine protease autotransporter, SPATE, Toxins, avian pathogenic *E. coli*, mouse infection, uropathogenic *E. coli*, poultry

## Abstract

Serine protease autotransporters of *Enterobacteriaceae* (SPATEs) are secreted proteins that contribute to virulence and function as proteases, toxins, adhesins, and/or immunomodulators. An extra-intestinal pathogenic *E. coli* (ExPEC) O1:K1 strain, QT598, isolated from a turkey, was shown to contain *vat, tsh*, and three uncharacterized SPATE-encoding genes. Uncharacterized SPATEs: Sha (*S*erine-protease *h*emagglutinin *a*utotransporter), TagB and TagC (*t*andem *a*utotransporter *g*enes *B* and *C*) were tested for activities including hemagglutination, autoaggregation, and cytotoxicity when expressed in *E. coli* K-12. Sha and TagB conferred autoaggregation and hemagglutination activities. TagB, TagC, and Sha all exhibited cytopathic effects on a bladder epithelial cell line. In QT598, *tagB* and *tagC* are tandemly encoded on a genomic island, and were present in 10% of UTI isolates and 4.7% of avian *E. coli*. Sha is encoded on a virulence plasmid and was present in 1% of UTI isolates and 20% of avian *E. coli*. To specifically examine the role of SPATEs for infection, the 5 SPATE genes were deleted from strain QT598 and tested for cytotoxicity. Loss of all five SPATEs abrogated the cytopathic effect on bladder epithelial cells, although derivatives producing any of the 5 SPATEs retained cytopathic activity. In mouse infections, *sha* gene-expression was up-regulated a mean of sixfold in the bladder compared to growth *in vitro*. Loss of either *tagBC* or *sha* did not reduce urinary tract colonization. Deletion of all 5 SPATEs, however, significantly reduced competitive colonization of the kidney supporting a cumulative role of SPATEs for QT598 in the mouse UTI model.

## Introduction

*Escherichia coli* is a common commensal of the gastrointestinal tract of mammals and birds, and is also a versatile pathogen associated with a variety of intestinal and extra-intestinal infections. Pathogenic *E. coli* belong to two main groups: intestinal pathogenic *E. coli*, and extra-intestinal pathogenic *E. coli* (ExPEC) [1,2]. Among ExPEC, the strains have been classified into pathotypes based on the sites of infection or the animal species they have infected, although these different ExPEC subgroups often share certain traits [3–8]. Such pathotypes include neonatal meningitis *E. coli* (NMEC), uropathogenic *E. coli* (UPEC), and avian pathogenic *E. coli* (APEC) [2,9,10]. Avian pathogenic *E. coli* (APEC) are a subset of ExPEC that cause respiratory infections and septicemia in poultry [4,10–12]. The genomes of a number of APEC strains and their virulence plasmids have been sequenced and share similarities to some human ExPEC isolates and their plasmids [13–18]. The plasticity of the *E. coli* genome has led to the emergence of numerous combinations of genes that can be encoded on genomic islands or harbored on plasmids that can contribute to fitness, adaptability, and virulence of a variety of ExPEC strains [19–21].

APEC and human ExPEC strains share multiple virulence factors that promote survival and colonization of the host during extraintestinal infections. These include fimbriae, iron acquisition systems, autotransporter (AT) proteins, capsular polysaccharides, O-antigens, toxins and secretion systems [1,2,9,11,12]. Most APEC strains also contain conjugative colicin V (ColV) or similar plasmids that encode multiple virulence genes that have been shown to contribute to virulence in poultry [11,22,23], and also to urinary tract infection or systemic infection in rodent models [6,24,25]. The shared battery of virulence genes and the close phylogenetic relatedness of some APEC and human ExPEC strains suggest that some APEC may be potential zoonotic pathogens for humans [6,7,25–28].

Among pathogenic *E. coli* virulence factors, AT proteins comprise a large family that falls into three main categories: SPATEs (Serine Protease Autotransporters of *Enterobacteriaceae*), trimeric AT proteins, and the self-associating autotransporters (SAATs), such as AIDA-1 and Antigen43 (Ag43) [29–31]. AT proteins are exported by the type V secretion system, which can be classified into 5 subgroups: Va for the monomeric autotransporters which includes SPATEs, Vb for the two-partner secretion system, Vc for the trimeric AT, Ve for the ATs that are homologous to both type Va and type Vb, and Vd for the intimins and invasins which have a reverse order of domains [32]. The export process of the AT may also require additional proteins such as the BAM and TAM assembly systems [33,34]. SPATEs consist of three specific domains: (i) a signal peptide which translocates the protein from cytoplasm to periplasm by the Sec-dependant pathway (ii) a functional passenger domain which contains a conserved serine protease motif (GDSGS), and (iii) a β-barrel domain which is localized in the outer membrane acting as a pore-forming domain that translocates the passenger domain [35]. SPATEs have been grouped into two main classes; class 1 SPATEs consist of cytotoxic proteins, whereas class 2 SPATEs represent immunomodulator proteins [32]. Certain SPATEs including the secreted autotransporter toxin (Sat), vacuolating autotransporter protein (Vat), temperature-sensitive hemagglutinin (Tsh), which has also been called hemoglobin protease (Hbp) [36], and protein involved in colonization (Pic) [37] have been previously reported in APEC and human ExPEC.

The SPATEs comprise a diverse group of autotransporter proteins that contribute to the virulence of pathogenic *E. coli* and *Shigella* spp., and other Enterobacteria [2,22,32,37–43]. Some SPATEs were shown to be important virulence factors in disseminated infection of ExPEC due to their proteolytic activity, which can promote the degradation of host cell substrates and elicit an inflammatory response [32,44]. In ExPEC, SPATE proteins have previously been characterized and have been shown to be associated with infections of both humans and other animals including poultry. SPATEs identified in uropathogenic *E. coli* include Sat [44], Vat [45,46] and PicU [41]. The *sat* gene encodes a vacuolating toxin and *sat* sequences were present in 55% of UPEC strains [40] but were not identified in a collection of APEC isolates [47]. PicU is homologous to the Pic protein identified in *Shigella* and enteroaggregative *E. coli* (EAEC) [37]. *PicU* was found in 22% of UPEC isolates [41] and 9% of APEC strains [47]. The Vat autotransporter was first discovered in APEC [45], was present in 60–70% of ExPEC from human infections [46,48] and 33% of APEC strains [47]. The Vat toxin was shown to contribute to virulence, respiratory infection, and cellulitis in broiler chickens [45]. Both *pic* and *vat* were shown to contribute to the fitness of UPEC in a mouse model of systemic infection [43]. Tsh was the first SPATE identified in *E. coli* [49] and was shown to contribute to the development of respiratory lesions in the air sacs of chickens [22]. The *tsh* gene is located on ColV-type plasmids, was present in 50% of APEC strains [47], is less commonly associated with human ExPEC, but can be associated with certain human ExPEC strains [18,50–52].

In this report, analysis of the genome sequence of an APEC O1 strain, QT598, revealed that it contained 5 distinct SPATEs. Three of these, two chromosomally encoded SPATE genes (we name *tagB* and *tagC*) and a novel plasmid-encoded SPATE gene *(sha*) have not been previously characterized. The remaining two SPATEs were the previously characterized Vat and Tsh proteins. Herein, we have characterized the three novel SPATEs, determined their prevalence among avian and human urinary tract isolates, and investigated the role of these SPATEs for cytotoxicity and in the colonization of the murine urinary tract.

## Results

### Genomic analysis identifies five predicted SPATEs encoded by E. coli strain QT598

Strain QT598 was initially isolated from an infected turkey poult in France as MT156 [53]. It is a phylogenetic group B2 strain belonging to serogroup O1, a common serogroup among ExPEC strains causing infections in both poultry and humans. This APEC strain was sequenced initially because it contains most of the known APEC-associated virulence genes and was previously found to be virulent in one-day-old chicks [54]. QT598 belongs to sequence type (ST) 1385. Other strains belonging to ST1385 include other APEC O1 isolates, a canine urinary isolate, and environmental isolates (http://enterobase.warwick.ac.uk/). Interestingly, ST1385 strains are related to other STs including ST91, which contains some strains from human extra-intestinal infections and *E. coli* F54, an O18:K1 human fecal isolate sharing many virulence genes found in ExPEC from neonatal meningitis [55].

**Figure 1.**
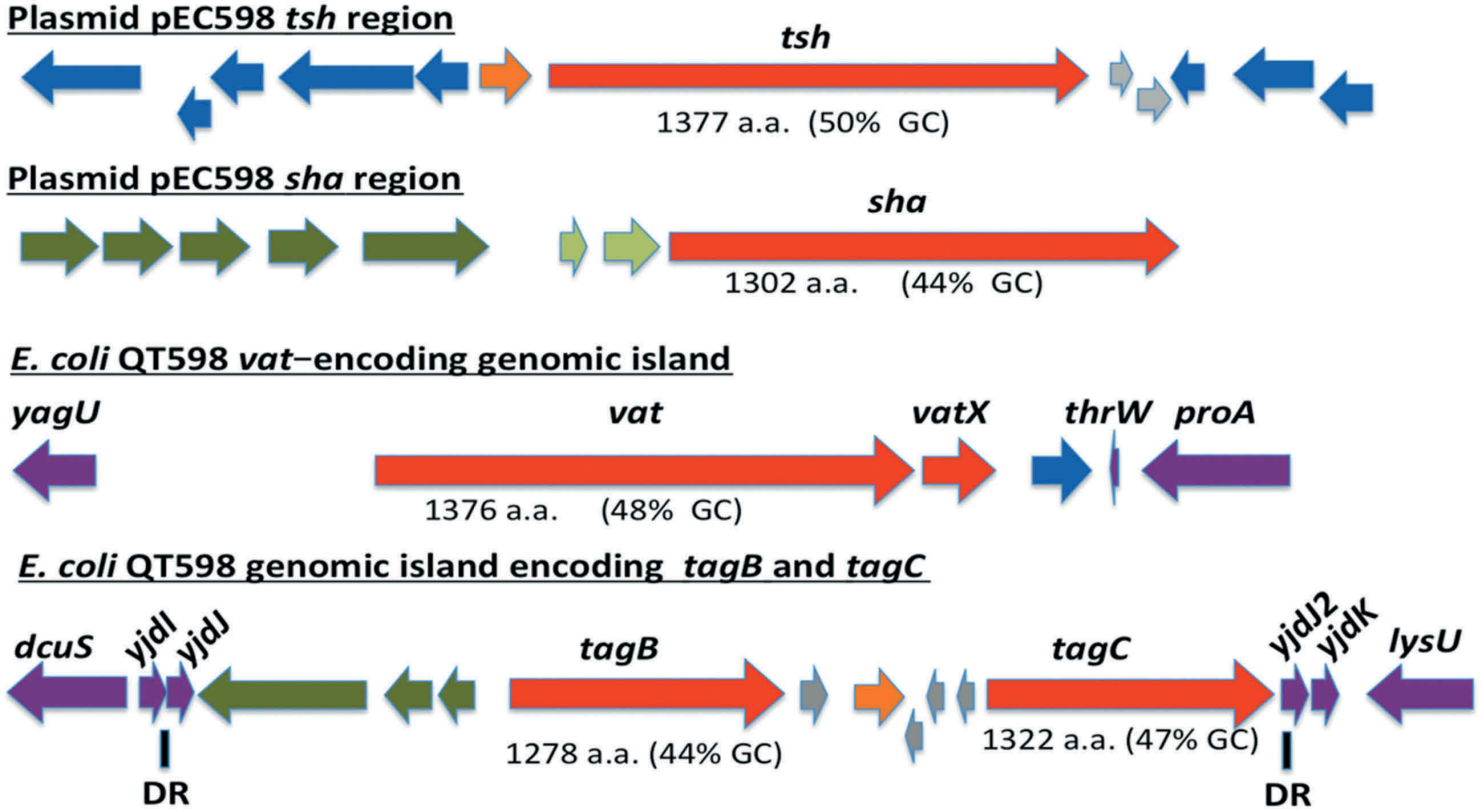
Regions containing the five SPATE-encoding genes in *E. coli* QT598. The *tsh* and *sha* genes are located on a ColV-type plasmid (pEC598). The *vat, tagB*, and *tagC* genes are located on genomic islands. Arrows indicate open reading frames (ORFs). SPATE encoding ORFs and regulatory gene *vatX* are in red. Predicted full amino acid lengths and GC content of the SPATE ORFs are indicated below arrows. Blue ORFs are related to insertion elements, integrases, or mobile elements. Dark green ORFs are predicted fimbrial proteins. Light green ORFS are predicted EAL-domain proteins. Grey ORFS are hypothetical uncharacterized ORFs. Orange ORFs are hypothetical regulatory proteins. Purple ORFs are genes conserved in *E. coli* K-12 that border the SPATE-encoding genomic regions. Direct repeats (DR) are indicated for the region containing the *tag* AT genes.

The genome of QT598 contains five SPATE-encoding sequences (Figure 1). Two of the SPATE genes, *tsh* and a novel SPATE which we have called *sha* (for ***s***erine-protease ***h***emaglutinin ***a***utotransporter), are located on a ColV-type plasmid (pEC598). The *vat* gene was also identified on a genomic island. Finally, a genomic region was identified containing two distinct SPATE-encoding sequences in close proximity to each other, which we have named *tagB* and *tagC* (for ***t***andem ***a***utotransporter ***g***enes *B* and *C*).

**Figure 2.**
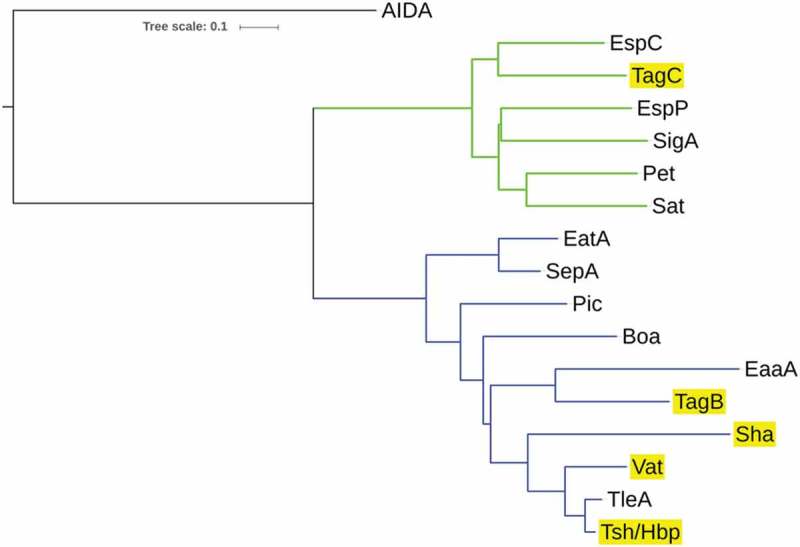
Phylogenetic analysis of new SPATEs identified in the QT598 genome. The evolutionary history of passenger domains of QT598 SPATEs (highlighted in yellow) as well as other characterized SPATEs was inferred using the Neighbor-Joining method [98]. The optimal tree with the sum of branch length = 8.78918031 is shown. The tree is drawn to scale, with branch lengths in the same units as those of the evolutionary distances used to infer the phylogenetic tree. The evolutionary distances were computed using the JTT matrix-based method [99] and are in the units of the number of amino acid substitutions per site. The analysis involved 17 amino acid sequences. All positions containing gaps and missing data were eliminated. There were a total of 723 positions in the final dataset. Evolutionary analyses were conducted in MEGA6 [85]. Multiple sequence alignment was performed by Clustal W, and the tree was constructed using the Mega6 software with PhyML/bootstrapping. A cluster of cytotoxic SPATEs (class 1) comprise the green branches, while immunomodulator SPATEs (class 2) are in blue branches. DNA regions encoding SPATE protein sequences are available in NCBI database as follows: EspC, GenBank Accession No. AAC44731, TagB and TagC, MH899681; EspP, NP_052685; SigA, AF200692; Pet, SJK83553; Sat, AAG30168; EatA, CAI79539, SepA, Z48219; Pic, ALT57188; Boa, AAW66606; EaaA, AAF63237; Sha, MH899684; Vat, MH899682; TleA, KF494347; Tsh/Hbp, MH899683.

The *tsh* open reading frame on plasmid pEC598 shares highest identity to *tsh* from plasmid pACN001-B (accession number KC853435.1) [56] and similar sequences in the NCBI database, differing in only 1 nucleotide, a Gly_1177_- Ser_1177_ substitution. Compared to the characterized Tsh (Hbp) proteins, Tsh from APEC strain χ7122 [22] and hemoglobin protease (Hbp) from ExPEC strain EB1 [36], Tsh_QT598_ contains 4 and 2 amino acid differences, respectively. In QT598, *tsh* is also flanked by sequences related to transposases and insertional sequences (Figure 1) that also flank *tsh* on other IncFII plasmids [22,36]. The *sha* gene is also located on pEC598 and has a 44% GC content. Sequence analysis of the *sha* gene revealed an open reading frame (ORF) of 3909 bp encoding a predicted precursor protein of 1302 amino acids with an N-terminal domain signal peptide (residues 1–51), a passenger domain (residues 52–1026) (predicted molecular mass of 105.7 kDa) containing a consensus serine protease motif ^256^GDSGS, and β-barrel domain (residues 1027–1302). Interestingly, the highly conserved SPATE cleavage site of two consecutive asparagines “EV**NN**LNK”, found between the passenger domain and the β-barrel of most SPATEs [57], is absent in Sha. Sha is more closely related to Tsh and Vat proteins than to other SPATEs (Figure 2). The global alignment of Sha with Tsh_QT598_ has 43% identity/56% similarity with 237 gaps, whereas the global alignment of Sha with Vat _QT598_ is 38% identity/52% similarity with 252 gaps.

The *vat* gene from QT598 is present on a genomic island that includes the *vatX* regulatory gene, and is located between the *E. coli* conserved genes *yagU* and *proA* adjacent to the *thrW*-tRNA gene (Figure 1). This is a conserved insertion site for *vat*-encoding genomic islands [58]. Vat_QT598_ is a predicted 1376 aa precursor with a single substitution (His_534_-Arg_534_) compared to Vat from UPEC strain CFT073 (accession number AAN78874.1). At least 41 predicted Vat protein sequences from different *E. coli* strains share an identical predicted Vat_QT598_ sequence, indicating it is a common allelic variant of Vat (Supplemental Table 2). These entries include sequences from strains isolated from fecal samples and infections of poultry and two human UTIs that are labeled as “Hbp” or “SepA” proteins in the databank.

The two new chromosomal-encoded SPATE genes were named *tagB* and *tagC*, **T**andem **a**utotransporter **g**enes (Tag) because of their tandem co-localization in the genome of QT598 as well as in various other *E. coli* strains such as: multidrug-resistant CTX-M-15-producing ST131 isolate *E. coli* JJ1886 (Accession number CP006784), porcine *E. coli* PCN033 (Accession number CP006632), and *E. coli* CI5 (Accession number CP011018). The *tagB* and *tagC* SPATE-encoding genes from QT598 are located on a genomic island between the *E. coli* conserved genes *yjdI* and *yjdK* (Figure 1). This genomic island has a mean GC content of 41%, which is considerably lower than the 50% GC of *E. coli*, suggesting horizontal gene transfer. This genomic region is also bordered by direct repeat (DR) sequences that correspond to duplication of *yjdJ* sequences bordering each side of the genomic island (Figure 1). Related genomic islands containing similar SPATE encoding genes at this insertion site are present in other *E. coli* genome sequences including antibiotic-resistant strains isolated from the urinary tract and other infections in humans (Supplemental Table 3). The predicted TagB and TagC proteins share the closest identity to the EaaA [59] and EspC [60], respectively (Figure 2). TagB shares 46% identity/63% similarity to EaaA with 84 gaps over its full length. TagC shares 60% identity/74% similarity to EspC with 22 gaps. TagB comprises a predicted signal peptide (residues 1–58), a passenger domain from residues 59–1006 (predicted molecular mass of 101 kDa) containing a consensus serine protease motif ^253^GDSGS, and a β-barrel domain from residues 1007–1283. TagC comprises a predicted signal peptide (residue 1–53), a passenger domain from residues 55–1032 (predicted molecular mass of 105.14 kDa) with a consensus serine protease motif ^250^GDSGS, and β-barrel domain ranging between 1033 and 1309 residues. Both TagB and TagC contain the conserved twin asparagine (N-N) residues in the linker domain connecting passenger and β-barrel domains, EIN^1006^NLNDRM and EVN^1032^NLNKRM, respectively.

#### Prevalence of new SPATE genes in human uropathogenic and avian pathogenic E. coli

To determine the distribution of the SPATE sequences among *E. coli* clinical isolates, the presence of these three new SPATE sequences as well as *vat, sat*, and *tsh* were detected by PCR in a collection of UPEC isolates from Guadeloupe (697 isolates) [61] and from avian pathogenic *E. coli* (299 isolates) [22]. For the UPEC isolates, *tagB* sequences were present in 70 isolates (10%), whereas *tagC* sequences were present in 80 isolates (11.5%). Interestingly, 96.8% (69/70) of the *tagB*-positive isolates were also *tagC*-positive. Furthermore, 68 of the *tagB* isolates belonged to phylogenetic group B2, with one isolate from group B1 and one untypable isolate. The 11 isolates that contained *tagC* but not *tagB* sequences belonged to groups other than B2: B1 (3 isolates), D (3 isolates), F (4 isolates), or A (1 isolate). *Sha* was the least common sequence, and was present in only 6 UTI isolates (0.9%), all of which belonged to group B2 and were also *vat*-positive. Five of the *sha*-positive strains also contained *tagB* and *tagC*, whereas *vat* and *sat* sequences were more common and found in 333 isolates (47.8%) and 217 isolates (31.1%), respectively. The *tsh* gene was present in 41 UPEC isolates (5.9%). In summary, in UPEC, *tagB* and *tagC* were found together in a subset of strains belonging to phylogenetic group B2, although some strains belonging to other phylogenetic groups were only *tagC* positive.

With regards to the APEC strains, *tagB* sequences were present in 14 isolates (4.7%) and *tagC* sequences were present in 21 isolates (7%). All 14 *tagB*-positive APEC were also *tagC*-positive, and 13/14 of these belonged to phylogenetic group B2. Among these, 10 strains belonged to serogroup O1, 1 was serogroup O78, and 3 were of undetermined serogroup. Interestingly, among the 299 APEC strains that were screened, comprised of 109 from chickens, 175 from turkeys, and 15 from ducks, all of the *tagB* or *tagC*-positive isolates were exclusively from infections in turkeys. Overall, similar to UPEC, *tagB*, and *tagC* were specifically present in a subset of APEC strains, mainly belonging to group B2, although some strains belonging to other phylogenetic groups only contained *tagC* sequences.

The *sha* sequences were present in 61 APEC strains (20%). The majority, 42 strains, belonged to phylogenetic group A, 11 strains belonged to group B1, 5 strains belonged to group B2, and 3 strains belonged to group D. Among these *sha*-containing strains, 35 belonged to serogroup O78, 3 were from serogroup O1, 2 strains each belonged to serogroups O11, O54, O21, and O8, and one belonged to serogroup O55. The remaining 12 strains were from undetermined serogroups. The *sha* gene is, therefore, clearly more prevalent among APEC than UPEC in the subset of strains we analyzed.

#### Cloning and production of SPATEs in culture supernatants

**Figure 3.**
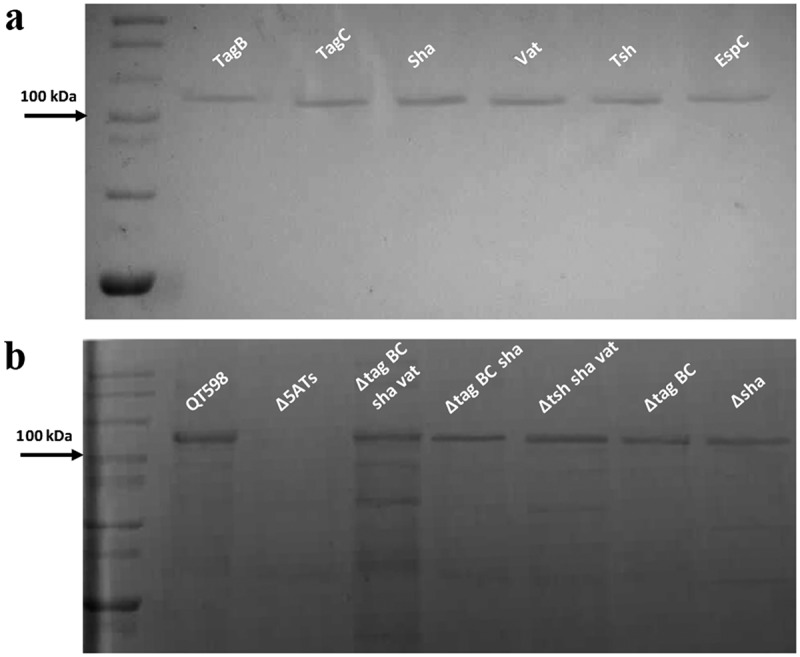
Detection of SPATE proteins by SDS-PAGE A. SDS-PAGE analysis of cloned SPATE genes. a. Clones expressing SPATE proteins were produced in the BL21 background with high-copy plasmid pBCsk+. Supernatants were filtered then concentrated through Amicon filters with 50 kDa cutoff. Samples containing 5 µg protein were migrated with protein marker (10–200 kDa) and stained with Coomassie blue (arrow represents 100 kDa size marker). b. Detection of SPATEs from supernatants of strain QT598 and various SPATE gene mutant derivatives. Supernatants from an overnight culture of the respective mutants were filtered, concentrated and run on SDS-polyacrylamide gels and stained with Coomassie blue to visualize proteins.

All five of the predicted SPATE-encoding genes and promoter regions were cloned to determine their expression and for use in a variety of phenotypic tests. Each of the five SPATE genes, when cloned into *E. coli* BL21, produced a high-molecular-weight protein (>100 kDa) in culture supernatants that corresponded to the expected product (Figure 3). In addition, derivatives of strain QT598 wherein these SPATE-encoding genes were inactivated were generated. Analysis of supernatant fractions of QT598, by SDS-PAGE, revealed the presence of SPATE proteins expressed under laboratory conditions, as demonstrated by visualization of bands with a high molecular mass (>100 kDa) secreted in the external milieu. By contrast, no such bands were observed in the supernatant extracts of the SPATE-free, Δ*5ATs*, derivative of QT598 (Figure 3). The purity of concentrated supernatant filtrate was also evaluated by silver staining (Supplemental Fig. S1). Protein bands of the newly identified SPATEs were extracted from gels and sampled by mass spectrometry for peptide analysis following trypsin digestion (Supplemental Fig. S2). For Sha, peptides corresponding to the mature secreted protein spanned from amino acids 52 to 1009. Despite not containing the twin asparagine (N-N) cleavage site present in most SPATEs, peptide profiles suggest the cleavage site from the β-barrel domain likely resides within the 1010–1020 region. This region contains two adjacent polar amino acids, Ser1015 and Asp1016, that may serve as the cleavage site. For TagB, peptide scans suggest that the mature secreted protein spans from amino acids 54 to 1006 based on the twin Asp1006-Asp1007 location. For TagC, peptide scans suggest that the mature secreted protein spans from at least amino acid 60 to 1026 with a predicted twin Asp1032-Asp1033 cleavage site. As expected, peptides corresponding to the predicted amino-terminal signal peptides and the carboxy-terminal predicted β-barrel domains of the Sha, TagB, and TagC SPATEs were not identified from peptide analysis of the secreted proteins (Supplemental Fig. S2).

#### Cleavage of oligopeptides by SPATE proteins

**Figure 4.**
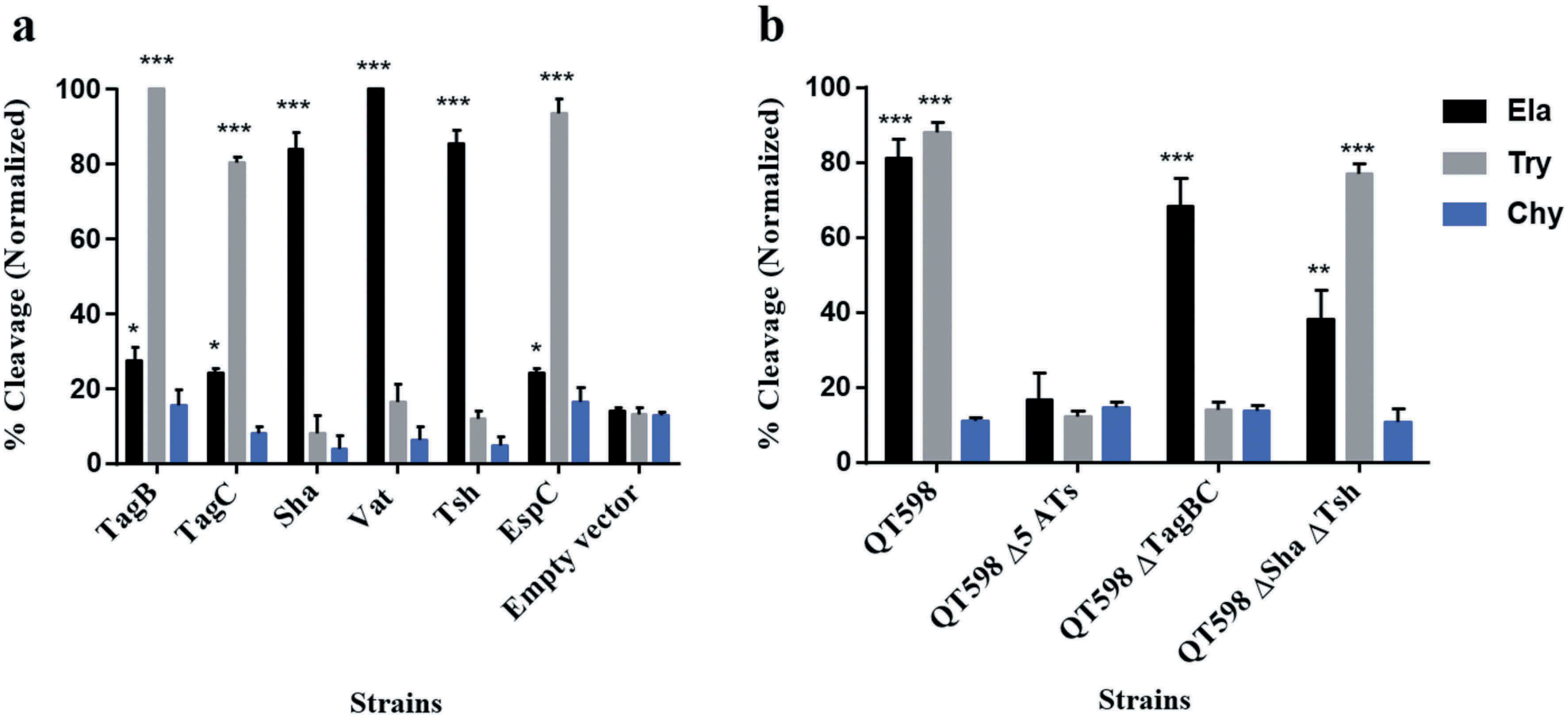
Oligopeptide cleavage profiles of SPATEs. a. Enzymatic activity of cloned SPATEs. Five μg of each SPATE-containing supernatant was incubated at 37°C for 3 h with 1mM of synthetic oligopeptide specifically recognized by the following enzymatic activities: Elastase (Ela)-(N-Suc-Ala-Ala-Ala-pNA); Trypsin (Try)-(N-Ben-L-arginine-pNA); or Chymotrypsin (Chy)-(N-Suc-Ala-Ala-Pro-Phe-pNA). Absorbance at 410nm was normalized to the maximum absorbance value. b. Enzymatic activity of supernatants from strain QT598 and SPATE gene mutant derivatives. Samples were tested as described above. Data are the means of three independent experiments, and error bars represent the standard errors of the means (* p < 0.05, **p < 0.01, ***p < 0.001 one-way ANOVA with multiple comparisons vs pBCsk+ (A) or QT598Δ*5AT*s (B)).

To determine the protease substrate cleavage specificity of the new SPATEs, we used synthetic polypeptides conjugated with *p*NA at the C-terminus. Purified proteins from supernatants of each SPATE were incubated with N-Succinyl-Ala-Ala-Ala-p-nitroanilide (elastase substrate), N-Benzoyl-L-arginine 4-nitroanilide (trypsin substrate) and N-succinyl-ala-ala-pro-phe-p-nitroanilide (chymotrypsin substrate) (Sigma-Aldrich, St. Louis, MO, USA). TagB and TagC demonstrated trypsin-like activity and efficiently cleaved N-Benzoyl-L-arginine 4-nitroanilide, similarly to the EspC protein (Figure 4(a)). By contrast, Sha demonstrated significant elastase-like activity toward N-Succinyl-Ala-Ala-Ala-p-nitroanilide, as did the Vat and Tsh proteins (Figure 4(a)). The cleavage activity of high-molecular-weight supernatant fractions from WT strain QT598 and SPATE mutant derivatives was also determined (Figure 4(b)). QT598 demonstrated both trypsin-like and elastase-like activity, whereas the Δ*5ATs* mutant had lost these activities. By contrast, a strain which had lost only *tagBC* demonstrated only elastase-like activity conferred by *vat, tsh*, and *sha* (Figure 4(b)). In addition, pre-incubation of these supernatants with PMSF eliminated or sharply inhibited oligo-peptide cleavage indicating the activity demonstrated was due to the SPATE proteins produced by the strains (Supplemental Fig. S3)

Multiple alignment of the new autotransporters with other SPATEs places TagC within the class 1, cytotoxic and enterotoxic SPATEs, along with the EspC SPATE from Enteropathogenic *E. coli* (EPEC) (Figure 2). EspC was previously shown to cleave spectrin, Factor V, pepsin and hemoglobin [42,62] and as with TagC and TagB, similarly demonstrated trypsin-like protease activity (Figure 4(a)). The SPATE sharing closest identity to TagB is the class 2 SPATE EaaA, identified from commensal *E. coli* ECOR-9 [59] (Figure 2). Sha shares more identity to class 2 SPATEs Tsh/Hbp (66% identity) [36,63], TleA (60% identity) [64] and Vat (56% identity) [45]. As such, Sha is likely to share other properties more similar to Tsh (adhesin, hemagglutinin) and Vat (cytotoxin), and the elastase-like substrate specificity of Sha, also shown for Tsh and Vat, is in line with this.

**Figure 5.**
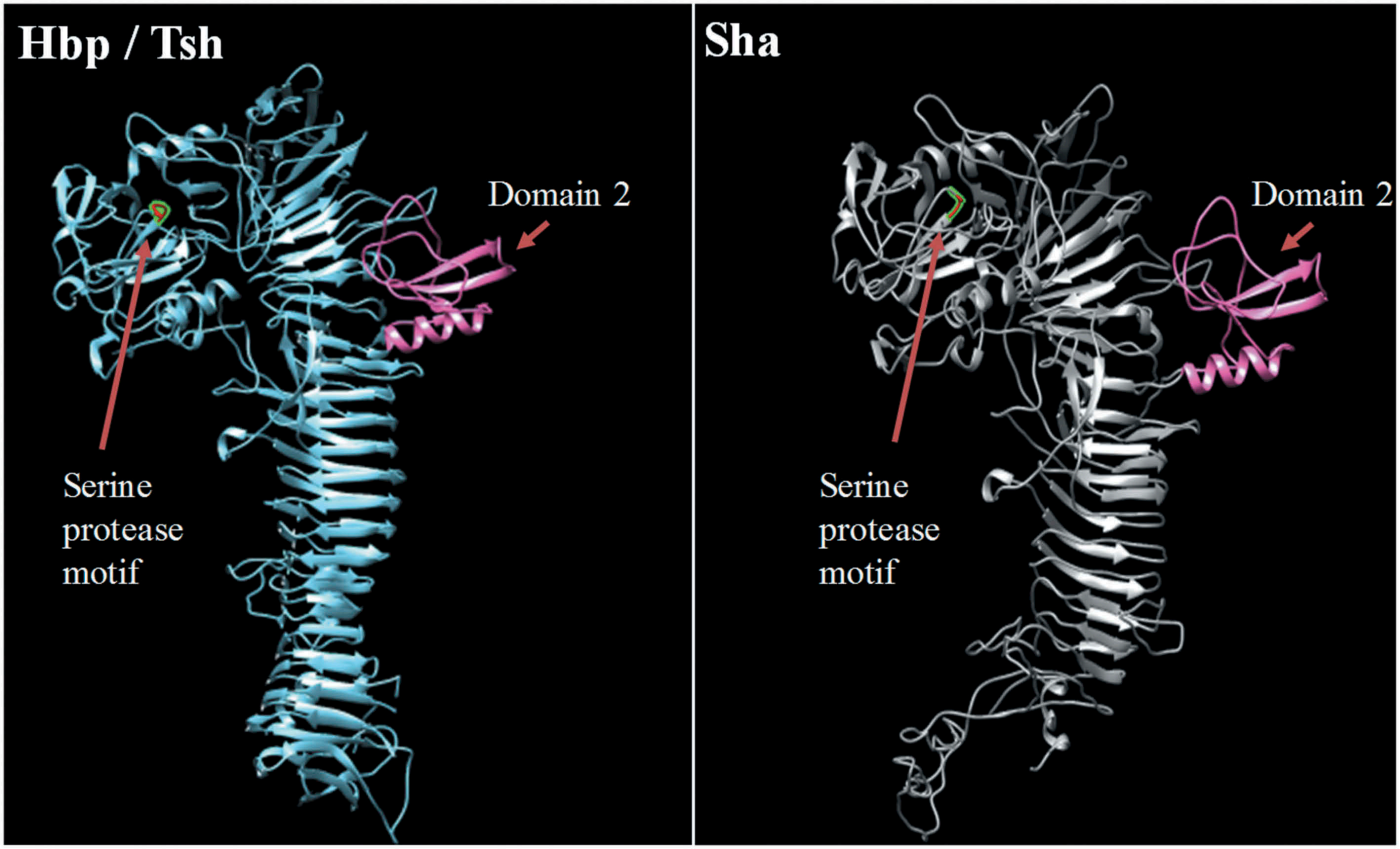
Stereo ribbon diagram showing the predicted three-dimensional structure of the Sha SPATE passenger domain derived from the Hbp/Tsh crystal structure. Crystal structure of the Heme-binding protein (Hbp) (PDB 1WXR), which is near identical to Tsh, was used to model a homologous structure based on alignment with the Sha protein sequence. The model was generated using the I-TASSER server with 100.0% confidence by the single highest scoring template. Sha is shown to harbor a conserved domain, domain 2 (shown in pink), which is characteristic of class 2 SPATEs.

To predict the 3D structure of the passenger domain of new SPATEs, we used the I-Tasser program to generate a 3D structure model and UCSF chimera to compare structures [65,66]. We found that the Sha protein is also predicted to contain the small domain 2 that was identified in Tsh/Hbp and was considered to be characteristic of class 2 SPATEs [32] (Figure 5). This domain was however absent in predicted models of TagB and TagC (Fig. S4).

#### Increased adherence to epithelial cells is mediated by SPATEs

**Figure 6.**
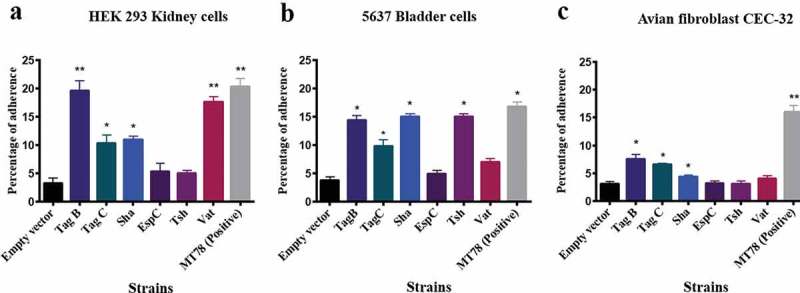
TagB, TagC, and Sha SPATEs promote adherence to the human kidney (HEK-293) and bladder (5637) epithelial, and avian fibroblast (CEC-32) cell lines. Cell monolayers were infected with *E. coli fim-*negative ORN172 expressing SPATE proteins at a multiplicity of infection (MOI) of 10 and incubated at 37°C at 5% CO_2_ for 2 h. Adherent bacteria were enumerated by plating on LB agar. Empty vector (pBCsk+) was used as a negative-control and APEC MT78 [80] as a positive control for adherence to cell lines. Data are the averages of three independent experiments. Error bars represent standard errors of the means. (*p < 0.05, **p < 0.01, ***p < 0.001 vs empty vector by one-way ANOVA).

Some AT proteins can promote adherence to host cells [31,67]. To investigate this with TagB, TagC, and Sha, the different SPATE encoding genes were cloned in the *fim*-negative *E. coli* K-12 strain ORN172 and tested for increased adherence to avian fibroblasts (CEC-32), human kidney (HEK-293) and bladder (5637) epithelial cells (Figure 6). Clones expressing either TagB, TagC, Sha, or Vat adhered significantly to kidney cells, whereas Tsh and EspC did not significantly increase adherence (Figure 6(a)). For the bladder cells, all SPATEs tested except for Vat and EspC significantly increased adherence (Figure 6(b)). By contrast, for avian fibroblasts, only TagB and TagC increased adherence (Figure 6(c)). These cell culture-based results suggest that TagB, TagC, and Sha, as well as Vat and Tsh, may contribute to host cell interactions in the urinary tract, and that TagBC may also contribute to adherence to tissues in poultry. Although some individual SPATEs were shown to increase adherence to host cells, loss of all 5 SPATEs did not reduce adherence of strain QT598 (Fig. S5).

#### Sha, TagB, Vat, and Tsh are hemagglutinins

Tsh has been previously shown to hemagglutinate chicken and sheep erythrocytes [22,49,68]. To assess whether other SPATEs also show hemagglutinin activity, we verified hemagglutination by the different SPATEs with erythrocytes from a variety of species. Interestingly, Sha, Tsh, and Vat all demonstrated hemagglutinin activities against sheep, bovine, pig, dog, chicken, turkey, rabbit, horse, and human blood (type O and A groups). In addition, TagB and TagC hemagglutinated sheep, bovine and pig erythrocytes, but not human, turkey, rabbit, dog, chicken, or horse erythrocytes (Table 1). However, the titer for TagC was very low for any erythrocytes tested and EspC demonstrated no hemagglutination.

**Table 2.** Strains and plasmids used in this study.

Strains	Characteristic(s)	References
ORN172	*thr-1 leuB thi-1*Δ*(argF-lac)U169 xyl-7 ara-13 mtl-2 gal-6 rpsL tonA2 supE44*Δ*(fimBEACDFGH)::Km pilG1*	[101]
BL21	*fhuA2 [lon] ompT gal [dcm] ΔhsdS*	New England Biolabs
QT1603	HB101 with AIDA-1 operon	[92]
QT598	APEC O1:K, serum resistant	[53]
MT78	APEC O2:K1	[80]
QT2799	*Serratia liquefaciens*	ATCC27592
QT4567	QT598 Δ*lacZYA*	This study
QT4726	QT598 Δ*tagBC::kan*, Km^R^	This study
QT5187	QT598Δ*tagBC*::FRT	This study
QT5188	QT598Δ*tagBC*Δ*vat::cat*, Cm^R^	This study
QT5189	QT598Δ*tagBC*Δ*vat::cat* Δ*sha::kan*, Cm^R^ Km^R^	This study
QT5182	Δ5*ATs* or QT598ΔtagBCΔvat:: *cat* Δ*sha:: kan* Δ*tsh::tetAR*(B) Cm^R^ Km^R^ Tc^R^	This study
QT5190	QT598Δ*sha:: kan* Δ*tsh:: tetAR*(B) Km^R^ Tc^R^	This study
QT5191	QT598Δ*tagBC* Δ*vat:: cat* Δ*tsh:: tetAR*(B) Cm^R^ Km^R^ Tc^R^	This study
QT5192	QT598Δ*sha:: kan* Km^R^	This study
QT5193	QT598Δ*tsh:: tetAR*(B) Tc^R^	This study
QT12	ORN172/pBCsk+	This study
QT4750	ORN172/pIJ551, (Expressing *vat*)	This study
QT4751	ORN172/pIJ552 (Expressing *tsh*)	This study
QT4767	ORN172/pIJ553 (Expressing *sha*)	This study
QT5194	BL21/pIJ548 (Expressing *tagB*)	This study
QT5195	BL21/pIJ549 (Expressing *tagC*)	This study
QT5197	BL21/pIJ550 (Expressing *espC)*	This study
QT5198	ORN172/pIJ548 (Expressing *tagB*)	This study
QT5199	ORN172/pIJ549 (Expressing *tagC*)	This study
QT5201	ORN172/pIJ550 (Expressing *espC)*	This study
QT5431	BL21/pIJ551 (Expressing *vat*)	This study
QT5432	BL21/pIJ552 (Expressing *tsh*)	This study
QT5433	BL21/pIJ553 (Expressing *sha*)	This study
**Plasmids**		
pKD3	Plasmid used for amplification of *cat* cassette	[89]
pKD4	Plasmid used for amplification of *kan* cassette	[89]
pKD13	Plasmid used for amplification of *kan* cassette	[89]
pKD46	λ Red plasmid; Amp^r^	[89]
pCP20	FLP recombinase, Amp^r^	[89]
pBCsk+	Cloning vector; Cm^r^	Stratagene, La Jolla, CA
pIJ548	pBCsk+::*tagB*	This study
pIJ549	pBCsk+::*tagC*	This study
pIJ550	pBCsk+::*espC*	This study
pIJ551	pBCsk+::*vat*	This study
pIJ552	pBCsk+::*tsh*	This study
pIJ553	pBCsk+::*sha*	This study

**Table 1.** Hemagglutination activities of different SPATEs.

Erythrocytes – Species (titer dilution)^a^									
SPATEs	Sheep	Bovine	Pig	Chicken	Turkey	Rabbit	Horse	Dog	Human
Vat	**6**	**5**	**4**	**3**	**3**	**7**	**4**	**7**	**7**
Tsh	**8**	**7**	**6**	**7**	**4**	**7**	**7**	**7**	**7**
Sha	**6**	**3**	**5**	**6**	**4**	**3**	**3**	**5**	**5**
TagB	**6**	**3**	**3**	-	-	-	-	-	-
TagC	**0–1**	**1**	**0–1**	-	**0–1**	-	-	-	-
EspC	-	-	-	-	-	-	-	-	-
pBCsk+ vector	-	-	-	-	-	-	-	-	-

**a-** Clones expressing SPATEs in *E. coli* ORN172 were adjusted to an O.D._600nm_ of 0.6 and then concentrated 100-fold. Samples were then diluted twofold in microwell plates containing final suspensions of 3% erythrocytes from different species. Titers are the average maximal dilution showing agglutination. Both human A and O blood gave similar titers. 0–1: little or no detectable agglutination. See Methods section for details.

#### TagB, TagC, and Sha mediate autoaggregation, but only Sha increases biofilm formation

**Figure 7.**
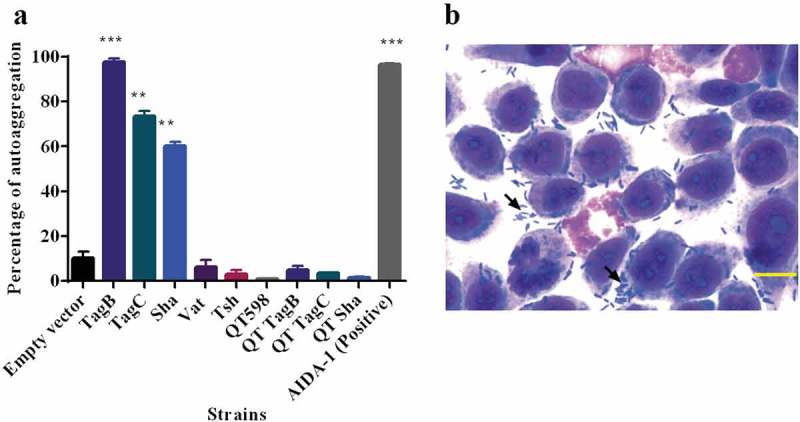
TagB, TagC, and Sha are autoaggregating proteins. a. Clones of *E. coli fim-*negative strain ORN172 expressing SPATE proteins were grown 18 h and adjusted to OD_600_ of 1.5 and left to rest at 4°C. Samples were taken at 1 cm from the top surface of the cultures after 3 h to determine the change in OD_600_. Assays were performed in triplicate, and the rate of autoaggregation was determined by the mean decrease in OD after 3 h. The autoaggregation phenotype was absent when plasmids expressing *tagB, tagC*, or *sha* were introduced into APEC strain QT598 (QT TagB, QT TagC, QT Sha respectively). Empty vector (pBCsk+) was used as a negative control and the AIDA-1 AT as a positive control for autoaggregation. Error bars represent standard errors of the means (* p < 0.05, **p < 0.01, ***p < 0.001 compared to empty vector using one-way ANOVA). b. Giemsa stain of the 5637 bladder cell line infected with a *tagB* expressing clone after 2 h demonstrates an aggregative adherence pattern to bladder cells (arrow head). A similar pattern was found for *tagC* and *sha* expressing clones (not shown here). Bar represents 50 µm.

Some AT proteins such as AIDA-1 and Ag43 mediate cell–cell interactions and autoaggregation, which can contribute to virulence and facilitate host cell adherence [69–71]. We observed that the absorbance of clones expressing Sha, TagB, and TagC dropped rapidly similar to the positive control AIDA-1(Figure 7(a)). An aggregative adherence pattern was also observed on interaction with bladder epithelial cell culture (Figure 7(b)). As these SPATEs demonstrated autoaggregation, we were interested to know if these plasmids could also confer autoaggregation to ExPEC QT598. However, the introduction of these plasmids did not lead to an autoaggregation phenotype in QT598 (Figure 7(a)).

**Figure 8.**
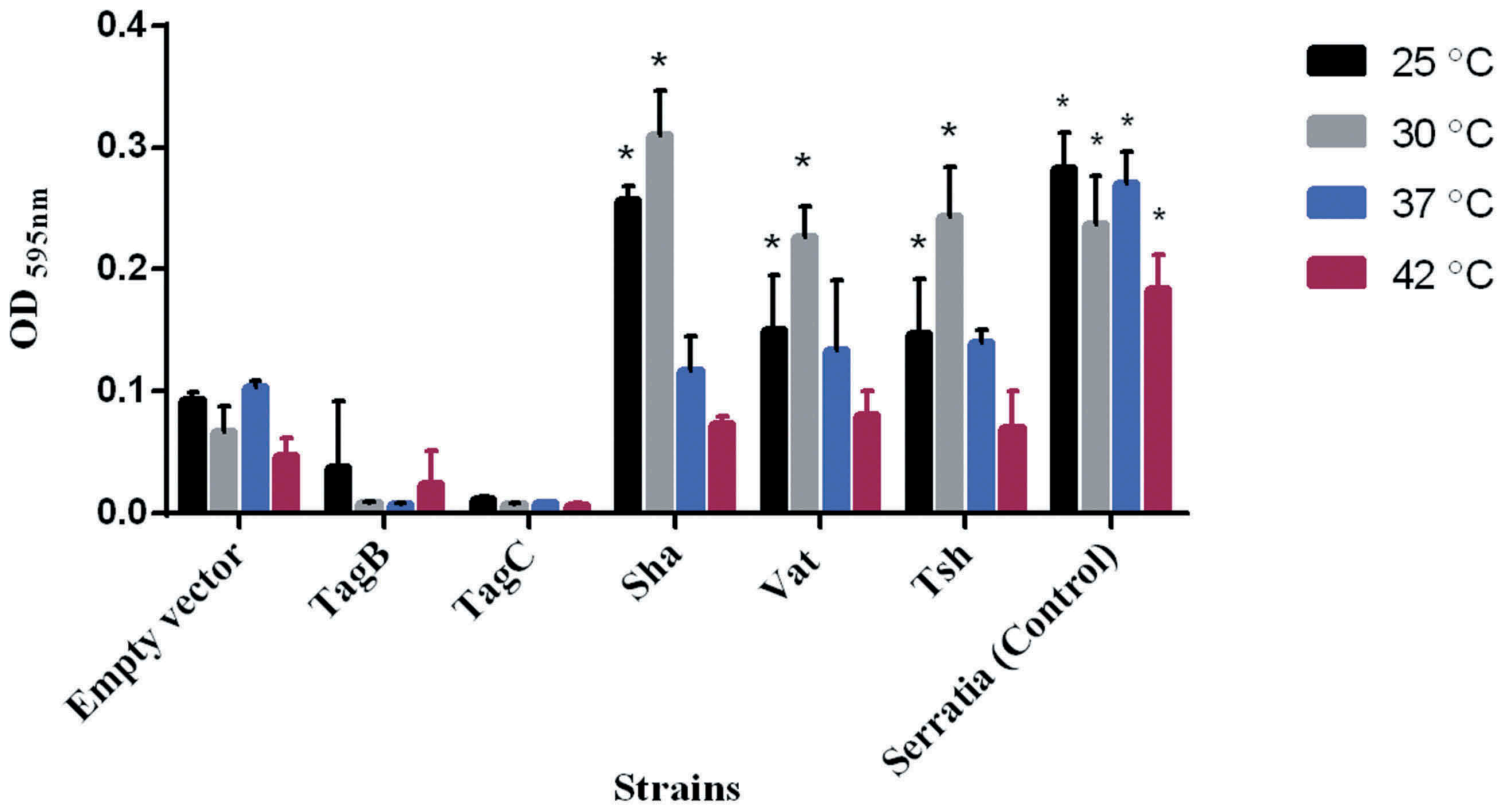
Sha, Vat, and Tsh promote biofilm formation. Clones of *E. coli fim-*negative strain ORN172 expressing SPATE proteins were grown at different temperatures (25°C, 30°C, 37°C, and 42°C) in polystyrene plate wells for 48 h and then stained with crystal violet. Remaining crystal violet after washing with acetone was measured as absorbance at 595 nm. Data are the means of three independent experiments, and error bars represent standard errors of the means. Empty vector (pBCsk+) was used as a negative control, and a string biofilm producing *Serratia* strain [100] served as a positive control for biofilm formation (* p < 0.05, **p < 0.01, ***p < 0.001 compared to empty vector using one-way ANOVA).

Proteins involved in autoaggregation can also increase biofilm formation [69]. We, therefore, checked biofilm forming capacity of these new SPATE clones at different temperatures (25°C, 30°C, 37°C, and 42°C). TagB and TagC did not increase biofilm formation. However, Sha, Vat, and Tsh significantly increased biofilm production at lower temperatures (25°C and 30°C), but no significant differences in biofilm production were observed at higher temperatures of 37°C and 42°C (Figure 8).

#### Assessment of the cytopathic effect of 5 different SPATEs on bladder cells

**Figure 9.**
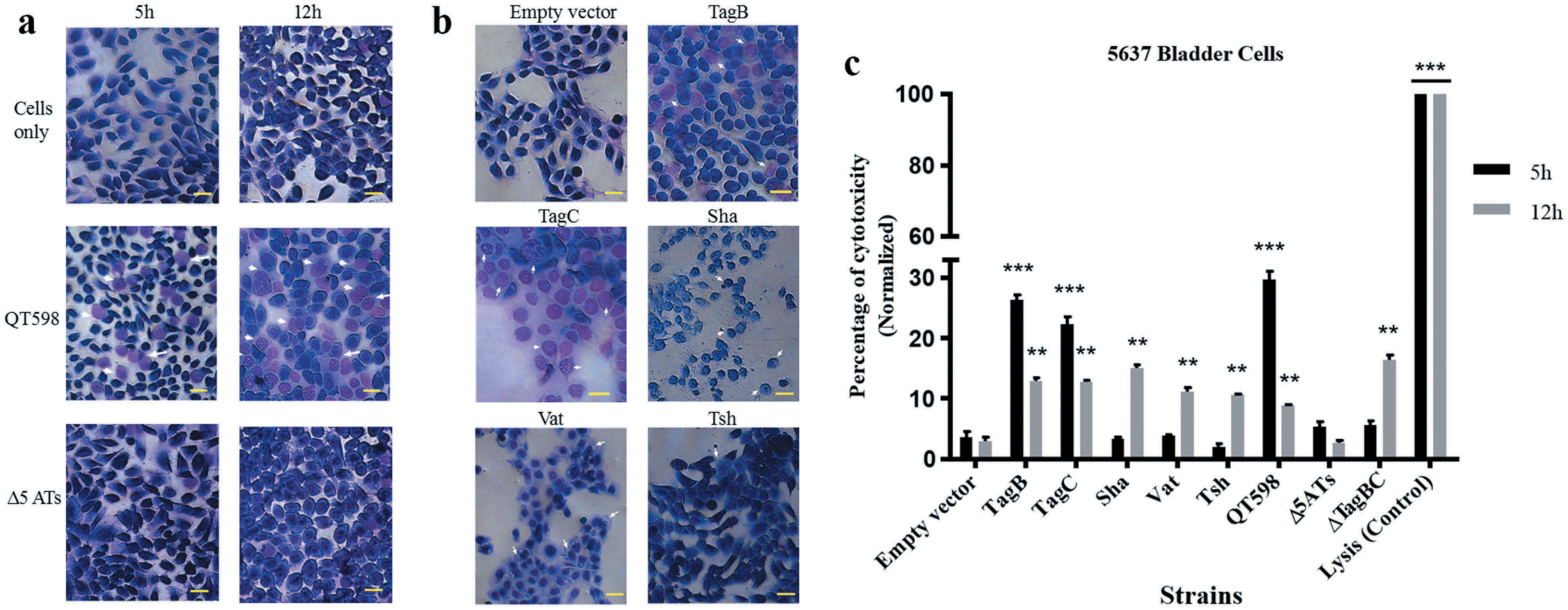
All five different SPATEs from strain QT598 induce cytopathic effects. a. Concentrated supernatants (30 μg of protein per well) from wild type QT598 as well SPATE free Δ5*AT*s were incubated with human bladder cell line 5637 for 5 h and 12 h. Cytopathic cells (arrowheads) were found in the cells treated with QT598 supernatant while there were no morphological changes in the cells treated with supernatant of Δ5*AT*s. b. Concentrated supernatants of *E. coli* BL21 pBCsk+ (30 μg of protein per well) clones overexpressing different SPATEs were incubated with monolayers of 5637 bladder cell lines for 12 h at 37°C. TagB, TagC, Sha, and Vat showed more cytopathic effect (arrowheads) compared to Tsh and empty vector. c. LDH release by 5637 cells after incubation with culture filtrates of different clones (30 μg of protein per well) expressing SPATEs or from supernatants from wild-type *E. coli* strain QT598 and Δ*5AT*s mutant derivatives with 5637 human bladder cells at 37°C for 5 h and 12 h. Empty vector (pBCsk+) was used as a negative control. Lysis solution was added as a positive control for maximum LDH release. Data are the means of three independent experiments, and error bars represent the standard errors of the means (* p < 0.05, **p < 0.01, ***p < 0.001 vs empty vector using one-way ANOVA). Bar represents 50 µm.

Since some SPATEs produce cytopathic activity on host cells, we assessed the cytopathic effect of extracts of supernatants of the different SPATEs as well as the supernatant of wild-type strain QT598 and SPATE-free Δ*5ATs* mutant, on the human bladder 5637 cell line. Incubation of SPATEs from concentrated filtered supernatant (Figure 3(b)) of strain QT598 with bladder cells triggered a cytopathic effect, characterized by the dissolution of cytoplasm and enlargement of the nucleus (Figure 9(a)), after 5 h of incubation. After 12 h, the majority of the cells were affected and showed similar morphological changes. These phenotypes were absent upon the treatment with the supernatant of the Δ*5AT* SPATE-free mutant or with culture media alone.

Further, we assessed the cytopathic effect of individual SPATEs that were from recombinant clones. Following 12 h of interaction, cells incubated with TagB or TagC proteins elicited distinct cytopathic changes including dissolution of cytoplasm, nuclear enlargement and vacuolation in the nucleus, cells treated with Sha were rounded, and Vat-treated cells showed numerous vacuoles within the cytoplasm (Figure 9(b)). By contrast, cells exposed to Tsh did not show distinct morphological changes. These results suggest that TagB, TagC, Sha and Vat proteases demonstrate cytopathic effects that alter bladder cell morphology, whereas Tsh was less cytopathic to this cell line. We further investigated the cytopathic effect of these SPATEs by measuring the release of lactate dehydrogenase (LDH) after 5 h and 12 h following exposure to supernatant extracts. The release of LDH after 5 h was demonstrated only following exposure to either TagB or TagC (Figure 9(c)). Interestingly, although LDH was not detected from samples exposed to Vat, Tsh and Sha after 5 h (Figure 9(c)), some LDH release was observed after 12 h of exposure, suggesting these SPATEs may demonstrate a delayed cytotoxic effect. Hence, all 5 SPATEs elicited some cytotoxicity that corresponded with cytotoxic effects that were observed in cells following Giemsa staining. Of further interest, the concentrated supernatant filtrates from ExPEC strain QT598 showed early toxicity comparable to the concentrated supernatant filtrates from *tagB* or *tagC* expressing clones. However, loss of *tagB* and *tagC* resulted in only a late onset cytotoxic effect at 12 h, and loss of all 5 SPATEs abrogated LDH release at either early or late time points (Figure 9(c)). Taken together, these results indicate TagB and TagC can mediate an early (5 h) cytotoxicity, whereas Vat, Tsh, and Sha mediate late onset (12 h) cytotoxicity, and that these 5 SPATES collectively mediate the overall cytotoxic effects of ExPEC QT598 on bladder epithelial cells.

#### Cumulative role of SPATEs for colonization during urinary infection in mice

**Figure 10.**
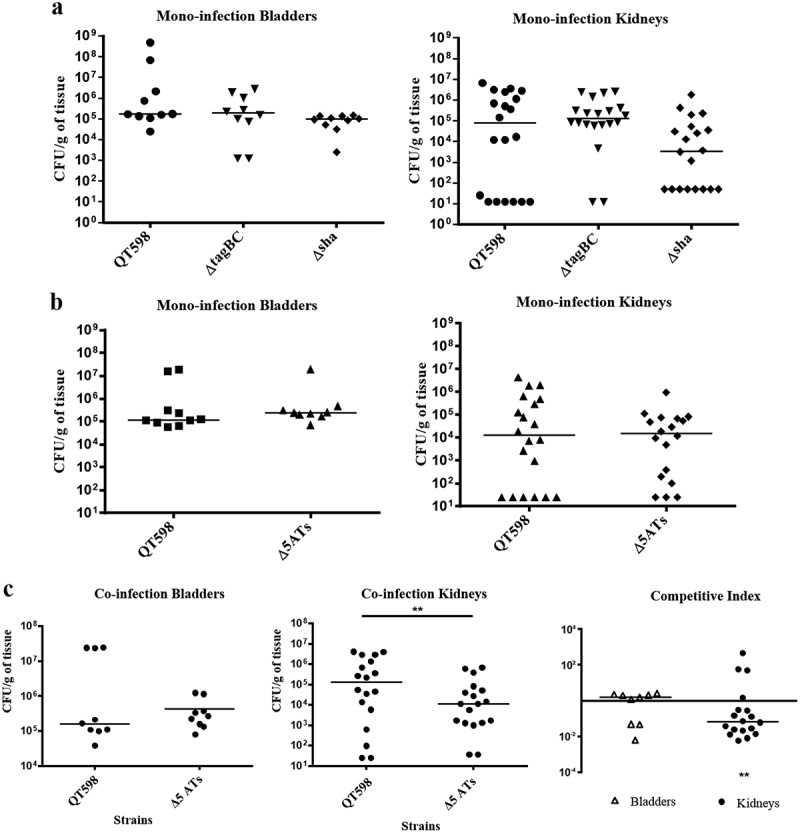
Role of SPATEs for *E. coli* QT598 in the murine model of ascending UTI. CBA/J mice were challenged transurethrally with QT598 and isogenic strains Δ*tagBC*, Δ*sha*, or Δ*5ATs* (wherein all 5 SPATE genes are inactivated). Mice were euthanized after 48 h, and bladder and kidneys were harvested for colony counts. a. Single-strain infections to compare wild-type strain QT598 to Δ*tagBC*, Δ*sha* mutants. There were no significant differences in bacterial numbers in either the bladders or kidneys. b. Single-strain infections to compare wild-type strain QT598 to the Δ*5ATs* mutant. Similarly, there were no significant differences in colonization observed. c. Co-infection experiments between the QT598Δ*lac* and the Δ*5ATs* mutant. The Δ*5ATs* mutant colonized the bladder at similar levels to the wild-type (Δ*lac*) strain; however, the Δ*5ATs* strain was outcompeted in the kidneys by over 10-fold (Data are means ± standard errors of the means of 10 mice (* p < 0.05, ** p < 0.01, Mann–Whitney Test).

In order to determine the potential role of SPATEs during UTI, we tested isogenic mutants with deletions of the *tagB, tagC*, and *sha* genes in a murine transurethral infection model. We observed no significant differences in colonization of the wild-type compared to *tagB, tagC* or *sha* knockout mutants (Figure 10). To determine the collective role of the SPATEs in UTI for QT598, we deleted all five SPATE encoding genes and did transurethral infections. We again observed no significant difference in the colonization of kidneys or bladder following single-strain infections. By contrast, when a co-infection model was used (1 mouse died after 24 h), the Δ*5ATs* mutant was significantly outcompeted by the wild-type by more than 10-fold in kidneys (p = 0.0037) (Figure 10)

#### Sha *gene expression is upregulated during infection in the mouse bladder*

**Figure 11.**
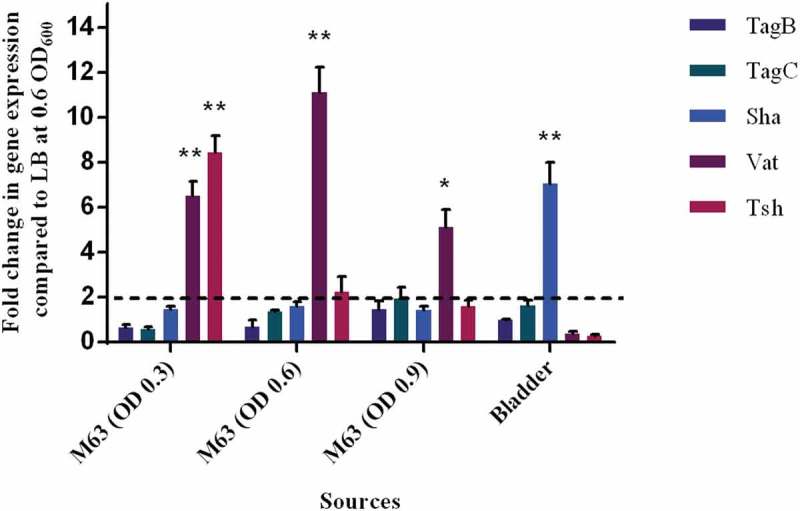
Differential expression of some SPATE genes occurs *in vitro* and in mouse bladder. qRT-PCR analysis of SPATE gene transcription from QT598 strain grown in different conditions. Growth in rich medium (LB) to OD_600_ of 0.6 was used as a standard and compared to growth in M63 minimal medium (with glycerol as carbon) at different growth phases (OD_600_ of 0.3, 0.6. and 0.9). RNAs were also extracted from infected mouse bladder. Transcription of *tsh* and *vat* genes were significantly increased in minimal medium. Further, the *sha* gene was shown to be significantly upregulated in the mouse bladder. (* p < 0.05, ** p < 0.01, error bars indicate standard deviations, Student *t-*test). Expression of other SPATE genes was tested under similar conditions. See methods section for details concerning the calculation of gene expression levels. Expressions of other SPATE genes were similar under all conditions tested. The dashed line corresponds to the cutoff for a significant difference in expression.

The level of expression of SPATE encoding genes from samples grown in different culture conditions as well as from infected mouse bladders was determined. All 5 SPATE genes were expressed in LB medium as well as in minimal M63-glycerol medium. Interestingly, the *vat* gene was upregulated by 10-fold in the minimal medium compared to LB. In bladder samples from infected mice, all the SPATE genes were detected and expressed during infection. Interestingly, the *sha* gene was upregulated 6-fold in the bladder (Figure 11). However, expression levels of the four other SPATE genes were not significantly different in bladders when compared to expression during culture in LB.

## Discussion

Some pathogenic *E. coli* produce multiple SPATEs, and this may provide a greater capacity to infect different host species or tissues. Having a combination of SPATEs may also allow a reserve of functions that may include both specific and redundant functions which may importantly be differentially regulated during infection or colonization. UPEC strain CFT073 has 10 autotransporter proteins including 3 SPATEs: Sat, Vat and PicU [41]. *Shigella flexneri* also contains 3 SPATEs: SepA, Pic and SigA [37,72,73]. Similarly, *Citrobacter rodentium*, a model for A/E lesions of EPEC and Enterohemorrhagic *E. coli* (EHEC) also produces 3 SPATEs [74]. The ExPEC strain QT598 we investigated in this report produces a total of 5 SPATEs including three previously uncharacterized members: Sha, TagB, and TagC. Each of the 5 SPATEs demonstrated individual as well as shared properties or functions. Sha, located on a ColV-type plasmid (pEC598), is a class 2 SPATE, and it is closely related to Tsh and Vat proteins. We found this new AT has a modified cleavage site lacking twin asparagine (NN) sites between the passenger domain and the β-barrel. This absence of a typical cleavage site was also seen in *rpeA* (*R*abbit-specific enteropathogenic *Escherichia coli* (REPEC) *p*lasmid-*e*ncoded *a*utotransporter) [75], indicating that the passenger domain is not cleaved from the outer membrane. However, in the case of Sha when cloned in BL21, a band was detected in polyacrylamide gel indicating that the modified cleavage site contributes to the separation of the passenger domain from the β-barrel.

TagB and TagC were found on a genomic island between the conserved *E. coli* genes *yjdI* and *yjdK*. Prevalence of the SPATE sequences among UPEC and APEC demonstrated that *tagB* and *tagC* sequences were present in at least 10% of the UPEC strains and that these genes were largely associated with strains belonging to phylogenetic group B2. Interestingly, genomic islands harboring *tagB* and *tagC* are also present in the genomes of numerous multi-resistant clinical isolates including members of the *E. coli* ST131 pandemic clone such as *E. coli* JJ1877 [76] and many other CTX-producing clinical isolates from urinary tract infections or sepsis (Supplemental Table 2). Further investigation into the potential role of these newly identified SPATE toxins for the virulence of such human ExPEC is therefore warranted.

Although *tagB* and *tagC* were less prevalent in APEC, the strains carrying those genes were mostly O1 strains belonging to phylogenetic group B2 and were all isolated from diseased turkeys. Although *sha* was only present in 1% of UPEC, it was present in 20% of APEC strains. As with Tsh, Sha is plasmid-encoded and these ColV-type plasmids are present in nearly all APEC but are less common in UPEC [77]. Similar phenotypes were observed for the Tsh, Vat, and Sha autotransporters, including hemagglutination, adherence, protease activity, biofilm formation, and cytopathic effects. As such, the role of these three SPATEs may also be cumulative for some APEC strains.

We assessed the cumulative role of SPATEs in APEC strain QT598 with cell cytotoxicity assays and infection experiments in the murine UTI model. It has been shown that certain APEC strains are highly similar to human ExPEC and can belong to the same clonal groups and contain similar virulence gene profiles [2,6,9,11,12,14,24,25]. Previous reports have also tested APEC strains in the mouse UTI model [78–81]. QT598 is a serogroup O1 strain, which is a common serogroup of both APEC and human ExPEC strains, and is clonally related to some human ExPEC, further supporting verification of the role of SPATEs for this strain in a UTI model. QT598 was initially isolated from a young turkey poult and was virulent by subcutaneous infection of 1-day-old chicks. We initially tested QT598 strain in a 3-week-old chicken air sac inoculation model. However, the strain only caused very limited disease and was rapidly cleared in this model. It is possible that the strain is only able to infect very young chicks or it may be more specific for infection of turkeys than chickens. It will be of interest to investigate this in the future.

By cloning each of the SPATE encoding genes in *E. coli* K-12, specific activities of individual SPATEs could be determined. Protease cleavage using oligopeptides demonstrated that Sha, as well as Tsh and Vat, demonstrated elastase-like activity, whereas TagB and TagC demonstrated trypsin-like activity, similar to the EspC autotransporter (Figure 4). Oligopeptide degradation was also observed in strain QT598 with TagB and TagC being required for trypsin-like activity, whereas the Class 2 SPATEs contributed to Elastase-like activity. As such, the combination of these SPATEs provides an expanded spectrum of protease activity. However, adding a serine protease inhibitor (PMSF) to the supernatant of the SPATEs neutralized their effect on the cleavage of these oligopeptides. Further, when the catalytic site of the ATs was mutated (serine was replaced by alanine) proteolytic activity was absent, indicating that the serine protease motif is important for the activity of these SPATEs (Supplemental Fig. S3). Previously, Tsh was shown to be proteolytic to substrates including mucin and factor V, whereas EspC could cleave other proteins such as pepsin and spectrin [62]. This suggests that the combination of SPATEs produced by QT598 can target multiple substrates. It will be of interest to more specifically investigate cleavage of different host substrates by the newly characterized SPATEs Sha, TagB, and TagC to try to identify their mechanisms of interaction with host cells. Adherence to host cell lines was also increased by the production of SPATEs. In particular, TagB, TagC, and Sha increased adherence to both human and avian epithelial cells, whereas Tsh only increased adherence to bladder cells, and Vat only increased adherence to kidney cells (Figure 6). Interestingly, in addition to promoting general adherence to host cells, TagB, TagC, and Sha also mediated bacterial aggregation, suggesting a self-associating phenotype similar to the AIDA-1 autotransporter (Figure 7). Although Tsh and Vat were less effective at general adherence to different epithelial cells, these SPATEs as well as Sha were effective hemagglutinins for erythrocytes of a variety of animal species and also demonstrated increased biofilm formation, whereas production of TagB and TagC only conferred limited hemagglutination activity (Table 2) and no increase in biofilm formation (Figure 8). It is interesting to note that some class 2 SPATEs have been shown to recognize a variety of glycans on leukocyte surfaces [82]. As similar carbohydrates may be present on erythrocyte surfaces, it is not surprising that Sha as well as Tsh and Vat autotransporters demonstrated extensive hemagglutination activity for a variety of erythrocytes. It will be of interest to further determine if these SPATEs can also recognize glycosylated surface receptors on either human or avian leukocytes that may alter host immune function.

Notably, some phenotypes such as autoaggregation, hemagglutination, and biofilm formation were only present when the genes were expressed in high-copy vectors but were absent in the clinical strain. Cloning in a higher copy vector provides a means to constitutively express proteins *in vitro*, whereas these proteins or systems may not always be expressed when in a single copy or on a native plasmid in the clinical strain under *in vitro* growth conditions. Also, in the wild-type clinical strain, there can be a great deal of redundancy of function with multiple proteins (both the autotransporters and other outer membrane proteins and fimbrial adhesins that may similarly mediate hemagglutination to different erythrocytes or adherence to host cells).

Interestingly all of the SPATEs demonstrated cytopathic effects on epithelial cells. However, the class 2 SPATEs, Vat, Tsh, and Sha, only caused delayed cell death after 12 h exposure, compared to the TagB and TagC SPATEs that demonstrated cytotoxicity within 5 h of interaction (Figure 9). Importantly, loss of all 5 of the SPATE encoding genes from QT598 was required to abrogate the cytopathic effect (Figure 9). However, the “slow acting” cytopathic effect may be due to a less directed or non-specific internalization such as pinocytosis for some of the SPATEs and since this effect only occurs after long-term 12-h exposure it may indicate that the cytotoxic capacity of these SPATEs may be limited and that the roles of such SPATEs may be more specifically linked to other protease functions such as mucinase activity or immunomodulatory roles as has been proposed as the main function of some of the Class-2 SPATEs. Similarly, a slow effect was seen in the case of EspC, when it was internalized slowly into the cells by pinocytosis, and no receptor was required for this process [83]. Further experiments are in progress to elucidate the cytopathic effects of Sha, TagB, and TagC.

Of further note, loss of all 5 SPATEs resulted in decreased fitness in the kidneys of infected mice (Figure 10), whereas loss of individual SPATEs did not have any reduction in virulence or fitness. This suggests that collectively these SPATEs can provide a selective advantage during kidney infection for QT598 in the murine UTI model. The levels of expression of the 5 SPATEs were different depending on the growth conditions – all 5 SPATEs were expressed in LB broth, mass spectrometry results have confirmed that Tsh (48% of coverage) and Vat (22% of coverage) proteins were highly expressed compared to the other SPATE proteins. In minimal M63-glycerol medium, the *vat* gene was upregulated by 10-fold compared to LB, and the *sha* gene was upregulated six-fold in infected bladder compared to culture in LB. These results indicate that SPATE-encoding genes can be subjected to environmental changes that influence their regulation. However, other than the *vat* gene [58], there is very limited information concerning identification of factors that regulate the expression of different SPATEs.

In conclusion, we investigated the role of three new SPATEs in an APEC strain, TagB, TagC, and Sha, which were present in some APEC and UPEC strains. These SPATEs may confer fitness and capabilities to infect multiple hosts. Our findings highlight the potential role of these proteins in virulence and show they significantly contribute to autoaggregation, hemagglutination, adherence to epithelial cell lines and also exhibit cytopathic effects. Furthermore, we have shown, in combination, all five ATs contribute to fitness and colonization of QT598 during urinary tract infection in the mouse model. In future studies, it will be of interest to determine mechanisms of action of the TagB, TagC, and Sha autotransporters, identify specific targets, and determine the effects of these SPATEs on host immune responses.

## Materials and methods

### Strains, media, and PCR screening

Strains and plasmids are listed in Table 2. The 697 *E. coli* strains isolated from urinary tract infections were clinical isolates from Guadeloupe. Strains were collected by laboratories or hospitals over a period of 17 months and included community or nosocomial urinary tract infection isolates [61]. The 299 APEC strains were previously described elsewhere [22]. MT156 (QT598) is an APEC serogroup O1 strain that was isolated from the liver of a young turkey poult [53]. *E. coli* DH5α, ORN172, and BL21 were used for gene cloning and phenotypic tests. Bacteria were grown at 37°C on solid or liquid Luria-Bertani medium (Alpha Bioscience, Baltimore, MD, USA) supplemented with the appropriate antibiotics when required at concentrations of 100µg/ml ampicillin, 30 µg/ml chloramphenicol, or 50 µg/ml of kanamycin. For mice infection studies, QT598 and mutant derivatives were grown in brain heart infusion broth (Alpha Bioscience). M63-glycerol minimal medium contained the following per liter: 5.3 g KH_2_PO_4_, 13.9 g K_2_HPO_4_ · 3H_2_O, and 2.0 g (NH_4_)_2_SO_4_. The pH was adjusted to 7.5 with KOH, and the medium was supplemented with 1 mM MgSO_4_, 1 mM CaCl_2_, 1 mM thiamine, and 0.6% (wt/vol) glycerol.

Multiplex PCR was performed to determine the prevalence of different ATs within clinical isolates using primers listed in Supplemental Table 1. PCR amplification was done in a 25 µl reaction mixture containing 10 mM of primers, 1X of Taq FroggaMix (FroggaBio, Toronto, ON, Canada), DNA templates and deionized water when necessary. To detect *sat, tsh, tagB* and *tagC* genes, the reaction mixture was placed in a thermocycler (Eppendorf, Mississauga, ON, Canada) and set for initial denaturation at 95°C for 2 min followed by 30 cycle of denaturation at 95°C for 30 sec, annealing at 58°C for 40 sec and extension at 72°C for 30 sec. A final extension step was added at 72°C for 5 min. In order to detect *sha* gene, PCR was set for initial denaturation at 95°C for 2 min, 35 cycle of denaturation (95°C, 30 sec), annealing (56°C, 40 sec) and extension (72°C, 1 min), and a final extension step at 72°C for 5 min. To detect *vat* gene, PCR was set for initial denaturation at 95°C for 2 min, 30 cycle of denaturation (95°C, 30 sec), annealing (60°C, 40 sec) and extension (72°C, 30 sec), and a final extension step at 72°C for 5 min. The phylogenetic group of strains was determined by multiplexed PCR as described [84]. Amplified samples were separated by electrophoresis on 0.8% agarose gel stained with gel stain (Civic Bioscience, Beloeil, QC, Canada) and DNA was visualized using Gel Doc (Syngene Chemi Genius, Frederick, MD, USA) at 400 nm.

### DNA and genetic manipulations

Plasmid DNA was extracted using the EZ DNA Miniprep kit (Bio Basic, Markham, ON, Canada). PCR products and DNA were purified using the EZ-10 Spin Column PCR Product Purification Kit (Bio Basic). DNA for SPATE-encoding genes was amplified using Q5 High Fidelity-DNA polymerase (New England Biolabs, Whitby, ON, Canada).

### Bioinformatic analysis

Presence of AT sequences *in silico* was determined in *E. coli* genomes available from the NCBI database by Blast analyses using the *tagB, tagC* and *sha* sequences from the QT598 genome. Phylogenetic analyses of the predicted full-length passenger domain sequence of each SPATE were performed by Clustal W, and the phylogenetic tree was constructed using PhyML/bootstrapping in MEGA6 [85]. The Conserved Domain Database (CDD) and SignalP were used to predict the three domains of the AT proteins [86,87]. The I-TASSER server and chimera were used for three-dimensional (3D) structure [65,66] visualization of the predicted Sha AT protein, and the Protein Data Bank (PDB) server was used to obtain the predicted 3-D structure of the Sha protein for comparison to Hbp [88]. Amino acid sequence comparisons were performed using Clone Manager Suite 7 (SciED, Denver, CO, USA) and online BLAST programs available from the National Center for Biotechnology Information (NCBI).

### Construction of plasmids

AT-encoding genes were amplified by PCR using specific primers (listed in Supplemental Table 2). The *tagB, tagC, sha, vat*, and *tsh* genes were amplified from QT598. The *espC* gene was amplified from EPEC strain E2348/69 (Accession number AF297061) [60]. To clone *tagB, tagC, espC, sha, tsh*, and *vat*, PCR products contained 15 bp extensions homologous to the pBCsk+ multi-cloning site. Linearized pBCsk+ digested with *Xho*I and *BamHI* was used to clone inserts by fusion reaction with the Quick-fusion cloning kit (Biotool, Houston, TX, USA). All the recombinant plasmids (pIJ548, pIJ549, pIJ550, pIJ551, pIJ552, pIJ553) were first transformed into *E. coli* DH5α then the plasmids were extracted using a Miniprep kit according to the manufacturer’s recommendations (Bio Basic) and transformed into *E. coli* BL21 for protein expression into culture supernatants and into the *fim*-negative *E. coli* strain ORN172 for other phenotypic assays.

### Mutagenesis of SPATE-encoding genes

Mutants were generated using the lambda red recombinase method [89]. The *tagBC* genes were first replaced with a kanamycin resistance cassette, from plasmid pKD13 with knock out primers: 2094 and 2095 (Supplemental Table 1). PCR products were electroporated into QT598 containing the lambda red recombinase-expressing plasmid pKD46. Deletion of the *tagB* and *tagC* was confirmed by PCR with screening primers (Supplemental Table 1). The kanamycin resistance cassette, flanked by FLP recombination target (FRT) sequences, was removed by the introduction of plasmid pCP20 expressing the FLP recombinase [90]. Then, the *vat* gene was replaced by a chloramphenicol resistance cassette amplified from pKD4 in the background QT598Δ*tagBC*::FRT (QT5187) using the same approach. Similarly, kanamycin resistance cassette amplified from plasmid pKD3 was used to replace the *sha* gene in QT598 Δ*tagBC* Δ*vat::cat* (QT5188). Finally, the *tsh* gene was replaced with a tetracycline-resistance cassette by allelic exchange as detailed in [22] in QT598 Δ*tagBC* Δ*vat::cat* Δ*sha::kan* (QT5189) generating the five SPATE genes, Δ5*ATs* mutant (QT598 Δ*tagBC* Δ*vat::cat* Δ*sha::kan* Δ*tsh::tetAR*(B)) (QT5182).

### Protein preparation and analysis

Culture supernatants, from LB broth cultures of *E. coli* BL21 expressing AT proteins were centrifuged at 7500 × g for 15 min at 4°C. Supernatants were filtered through 0.22 μm filters and concentrated through 50 kDa Amicon filters (Millipore Sigma, St. Louis, MO, USA). Protein concentrations were determined using the Pierce Coomassie Plus Assay Reagent kit (Thermo Fisher Scientific, St. Laurent, QC, Canada) and proteins were visualized on SDS-PAGE by Coomassie blue as well as silver staining and identified based on the protein markers (10–200 kDa) (Bio Basic). To identify the proteins, bands were excised from denatured gels. Protein digestion by the trypsin, peptide labeling, and mass spectrometry analyses was performed by the proteomics platform of the Institut de Recherche en Immunologie et en Cancérologie (IRIC) of the Université de Montréal (Montréal, QC, Canada). The data were visualized and analyzed using Scaffold 4 Proteomics software.

### Oligopeptide cleavage assays

Synthetic peptide cleavage was performed as previously described in [91] with slight modifications. Briefly, 5 µg/ml of each SPATES was added to 200 μl of three different pNA-conjugated oligopeptides: N-Succinyl-Ala-Ala-Ala-p-nitroanilide, N-Benzoyl-L-arginine 4-nitroanilide and N-succinyl-ala-ala-pro-phe-p-nitroanilide (Millipore Sigma) at 1 mM concentration in a buffer containing 0.2 M NaCl, 0.01 mMZnSO_4_, 0.1 M MOPS (3-(N-morpholino) propanesulfonic acid), pH 7.3 were incubated at 37°C for 3 h and absorbance readings were determined at 410 nm. Readings were normalized to the maximum absorbance of positive control. All reactions were performed in triplicate.

### Autoaggregation

The autoaggregation test was carried out as described before [92]. Briefly, overnight cultures were adjusted to an OD_600_ of 1.5. A volume of 10 ml of each culture was placed in sterile 20 ml glass tubes. Tubes were then vortexed for 5 s then left at 4°C for 3 h. Samples 1 cm from the top were taken, and the percentage of change in OD_600_ was used as the value for autoaggregation.

### Hemagglutination tests

Hemagglutination in microtiter plates was performed as described by [93] with some modifications. Human, chicken, turkey, pig, bovine, canine, rabbit, horse and sheep red blood cells (RBCs) were washed and resuspended in PBS at a final concentration of 3%. The *E. coli fim*-negative K-12 strain ORN172 expressing different SPATEs was grown overnight at 37°C in LB medium, harvested and adjusted to an optical density (O.D._600_) of 60. Suspensions were serially diluted in 96-well round bottom plates containing 20 µl of PBS mixed with 20 µl of 3% red blood cells and incubated for 30 min at 4°C.

### Biofilm assay

Biofilm formation on polystyrene surfaces was assessed in 96-well plates as previously described [67]. Strains were grown at various temperatures (25°C, 30°C, 37°C, and 42°C) for 48 h under static conditions in LB medium. Wells were washed and stained with 0.1% crystal violet (Millipore Sigma) for 15 min, then 200 µl of ethanol-acetone (80:20) solution was added, followed by measuring at an optical density at OD_595_.

### Adherence assays

The 5637 human bladder cells (ATCC HTB-9), human embryonic kidney cells HEK-293 (ATCC® CRL-1573™), and the avian fibroblast cell line (CEC-32) were used to determine adherence of *E. coli* ORN172 expressing different SPATEs as described before [94]. The 5637 cells were maintained in RPMI 1640 (Thermo Fisher Scientific) supplemented with 10% heat-inactivated fetal bovine serum (FBS) at 37°C in 5% CO_2,_ and 2 × 10^5^ cells/well were seeded into 24-well cell culture plates. Cell lines were washed twice with phosphate-buffered saline (pH 7.2) and then incubated at a multiplicity of infection (MOI) of 10 at 37°C for 2 h in RPMI 1640 medium with 10% FBS. Non-adherent bacteria were removed by washing with PBS three times. Cells were then exposed to 1% Triton X-100 for 5 min, and serial dilutions were plated on LB agar plus an antibiotic. For HEK-293 cells, Eagle’s Minimum Essential Medium supplemented with 10% of FBS was used. For CEC-32 cells Dulbecco’s Modified Eagle’s medium supplemented with 10% of FBS was used. The adherence assays were done in triplicate for each sample.

### Determination of cytopathic effects

The 5637 cells were maintained in RPMI 1640 medium (Thermo Fisher Scientific) supplemented with 10% heat-inactivated FBS at 37°C in 5% CO_2,_ and 2 × 10^5^ cells/well were seeded into eight-well chamber slides (Thermo Fisher Scientific) and allowed to grow to 75% confluence. Thirty μg/ml of each SPATE (final concentration) was added directly to monolayers and incubated for 5 h or 12 h at 37°C with 5% CO_2_. Cells were then washed twice with PBS (phosphate-buffered saline), fixed with 70% methanol, and stained with Giemsa stain.

### Lactate dehydrogenase (LDH) release assay

Culture supernatants were incubated with monolayers of 5637 cells in RPMI 1640 supplemented with 10% heat-inactivated FBS at 37°C in 5% CO_2_ for up to 12 h. Release of LDH was quantified by CytoTox 96® Non-Radioactive Cytotoxicity Assay kit (Promega, Madison, WI, USA) at 5 h and 12 h. A lysis solution (provided in the kit) was added to the non-infected cells to generate the maximum LDH release control from lysed cells.

### Ascending urinary tract infection in mice

For single strain infections with the wild-type parent QT598 and isogenic SPATE mutants QT598Δ*tagBC* and QT598Δ*sha*, 25 μl (10^9^ CFU) were tested in an ascending UTI model adapted from [95] with 10 mice in each group. Similarly, a murine ascending UTI model with 10 mice in each group was used for co-infection, in which a virulent Δ*lacZYA* derivative of QT598 was co-infected with the Δ*5ATs* strain, a QT598-derivative lacking all 5 SPATE-encoding genes. Twenty-five μl (10^9^ CFU) of a mixed culture containing equal amounts of each strain were inoculated through a catheter in six-week-old CBA/J female mice. Mice were euthanized after 48 h, and bladders and kidneys were harvested aseptically, homogenized, diluted and plated on MacConkey agar plates. Bladder samples were frozen at −80°C in TRIzol® reagent (Thermo Fisher Scientific) for RNA extraction.

### *qRT-PCR analysis of SPATE gene expression* in vivo *and* in vitro

Expression of the 5 SPATE-encoding genes was determined after growth in LB medium, minimal M63 medium, and from infected mouse bladders. Total RNAs from bacterial samples were extracted in the EZ-10 Spin Column Total RNA Miniprep Kit (Bio Basic) as described elsewhere [96]. Briefly, to extract RNA from infected bladder, samples were homogenized with TRIzol® reagent (Thermo Fisher Scientific), centrifuged for 30 sec at 12,000 × g, the supernatant was incubated with ethanol (95–100%) and transferred into Zymo-Spin™ IICR Column to extract RNA using Direct-zol RNA Miniprep kit (Zymo Research, Irvine, CA, USA) according to the manufacturer’s recommendations. All RNA samples were treated with TURBO DNase (Thermo Fisher Scientific). PCR (35 cycles) was used to verify DNA contamination. cDNAs were synthesized by using the Iscript^TM^ Reverse transcription supermix according to the manufacturer’s protocol (Bio-Rad Life Science, Mississauga, ON, Canada). The RNA polymerase sigma factor *rpoD* gene was used as a housekeeping control. Primers designed to amplify *rpoD, tagB, tagC, vat, tsh,* and *sha* (Supplemental Table 1) were used. For each sample, 50 ng of cDNA and 100 nM concentrations of each primer set were mixed with 10 μl of SsoFast Evagreen supermix (Bio-Rad Life Science) per well. Assays were performed in triplicate on a Corbett Rotorgene (Thermo Fisher Scientific) instrument. All data were normalized to *rpoD* expression levels. Melting-curve analysis was verified to differentiate accumulation of Evagreen-bound DNA and determine that signal was gene-amplification specific and not due to the primer-dimer formation. The data were analyzed by the 2^−ΔΔ*CT*^ method [97].

### Statistical analyses

Experimental data were expressed as a mean ± standard error of the mean (SEM) in each group. The means of groups were combined and analyzed by a two-tailed Student *t*-test for pairwise comparisons and analysis of variance (ANOVA) to compare means of more than two populations. For mouse infection experiments, the Mann–Whitney test was used to compare the samples by pairs, and the Kruskal–Wallis test was used to compare groups. A *P* value of <0.05 was considered statistically significant. All data were analyzed with the Graph Pad Prism 7 software (GraphPad Software, San Diego, CA, USA).

### Ethics statement

The protocol for mice urinary tract infection was approved by the animal ethics evaluation committee – *Comité Institutionel de Protection des Animaux* (CIPA No 1608–02) of the INRS-Institut Armand-Frappier.

### Nucleotide accession numbers

Sections of the genome of *E. coli* strain QT598 containing the five different SPATE-encoding genes were submitted to NCBI Genbank from the analysis of whole-genome survey sequence. The corresponding accession numbers are *tsh* (MH899683), *sha* (MH899684), *vat* (MH899682), and *tagB* and *tagC* (MH899681) genetic regions.
