# Supplementary material for: Three new serine-protease autotransporters of Enterobacteriaceae (SPATEs) from extra-intestinal pathogenic Escherichia coli and combined role of SPATEs for cytotoxicity and colonization of the mouse kidney
Source: Virulence. 2019 Jun 14;10(1):568–87. doi: 10.1080/21505594.2019.1624102 (PMC6592367; doi:10.1080/21505594.2019.1624102)
Supplement: Supplemental Material [file kvir-10-01-1624102-s001.zip › Supplemetal_Tables.docx]

**Supplemental Table 1.**

**List of *E. coli* strains and accession numbers for Vat proteins identical to Vat_QT598_**

| **Strain/isolate** | **Accession number** | **Information or source** |
| --- | --- | --- |
| KTE28 | [ELC53090.1](https://www.ncbi.nlm.nih.gov/protein/ELC53090.1?report=genbank&log$=protalign&blast_rank=1&RID=YKTNX4XS014) | UTI |
| UMEA 4076-1 | [ERA51660.1](https://www.ncbi.nlm.nih.gov/protein/ERA51660.1?report=genbank&log$=protalign&blast_rank=1&RID=YKTNX4XS014) | UTI |
| 004PP2015 | [OCS73020.1](https://www.ncbi.nlm.nih.gov/protein/OCS73020.1?report=genbank&log$=protalign&blast_rank=1&RID=YKTNX4XS014) | Turkey colibacillosis |
| FEX675 | [PPE49807.1](https://www.ncbi.nlm.nih.gov/protein/PPE49807.1?report=genbank&log$=protalign&blast_rank=1&RID=YKTNX4XS014) | Retail chicken meat potential ExPEC |
| VRES0524 | [SQW13100.1](https://www.ncbi.nlm.nih.gov/protein/SQW13100.1?report=genbank&log$=protalign&blast_rank=1&RID=YKTNX4XS014) | Turkey feces |
| VRES0524 | [SQV09766.1](https://www.ncbi.nlm.nih.gov/protein/SQV09766.1?report=genbank&log$=protalign&blast_rank=1&RID=YKTNX4XS014) | Turkey feces |
| VRES0540 | [SQX23694.1](https://www.ncbi.nlm.nih.gov/protein/SQX23694.1?report=genbank&log$=protalign&blast_rank=1&RID=YKTNX4XS014) | Turkey feces |
| VRES0561 | [SQW81848.1](https://www.ncbi.nlm.nih.gov/protein/SQW81848.1?report=genbank&log$=protalign&blast_rank=1&RID=YKTNX4XS014) | Turkey feces |
| VRES0558 | [SQW95192.1](https://www.ncbi.nlm.nih.gov/protein/SQW95192.1?report=genbank&log$=protalign&blast_rank=1&RID=YKTNX4XS014) | Turkey feces |
| VRES0563 | [SQX99582.1](https://www.ncbi.nlm.nih.gov/protein/SQX99582.1?report=genbank&log$=protalign&blast_rank=1&RID=YKTNX4XS014) | Turkey feces |
| VRES0557 | [SQZ03425.1](https://www.ncbi.nlm.nih.gov/protein/SQZ03425.1?report=genbank&log$=protalign&blast_rank=1&RID=YKTNX4XS014) | Turkey feces |
| VRES0564 | [SRZ55961.1](https://www.ncbi.nlm.nih.gov/protein/SRZ55961.1?report=genbank&log$=protalign&blast_rank=1&RID=YKTNX4XS014) | Turkey feces |
| VRES0569 | [SQX13605.1](https://www.ncbi.nlm.nih.gov/protein/SQX13605.1?report=genbank&log$=protalign&blast_rank=1&RID=YKTNX4XS014) | Turkey feces |
| VRES0574 | [SQX78012.1](https://www.ncbi.nlm.nih.gov/protein/SQX78012.1?report=genbank&log$=protalign&blast_rank=1&RID=YKTNX4XS014) | Turkey feces |
| VRES0567 | [SQX85253.1](https://www.ncbi.nlm.nih.gov/protein/SQX85253.1?report=genbank&log$=protalign&blast_rank=1&RID=YKTNX4XS014) | Turkey feces |
| VRES0560 | [SRB39602.1](https://www.ncbi.nlm.nih.gov/protein/SRB39602.1?report=genbank&log$=protalign&blast_rank=1&RID=YKTNX4XS014) | Turkey feces |
| VRES0571 | [SQX68131.1](https://www.ncbi.nlm.nih.gov/protein/SQX68131.1?report=genbank&log$=protalign&blast_rank=1&RID=YKTNX4XS014) | Turkey feces |
| VRES0528 | [SRB74388.1](https://www.ncbi.nlm.nih.gov/protein/SRB74388.1?report=genbank&log$=protalign&blast_rank=1&RID=YKTNX4XS014) | Turkey feces |
| VRES0550 | [SRA14460.1](https://www.ncbi.nlm.nih.gov/protein/SRA14460.1?report=genbank&log$=protalign&blast_rank=1&RID=YKTNX4XS014) | Turkey feces |
| VRES0527 | [SQV24988.1](https://www.ncbi.nlm.nih.gov/protein/SQV24988.1?report=genbank&log$=protalign&blast_rank=1&RID=YKTNX4XS014) | Turkey feces |
| VRES0541 | [SQV90344.1](https://www.ncbi.nlm.nih.gov/protein/SQV90344.1?report=genbank&log$=protalign&blast_rank=1&RID=YKTNX4XS014) | Turkey feces |
| VRES0531 | [SRZ02671.1](https://www.ncbi.nlm.nih.gov/protein/SRZ02671.1?report=genbank&log$=protalign&blast_rank=1&RID=YKTNX4XS014) | Turkey feces |
| VRES0529 | [SQW37114.1](https://www.ncbi.nlm.nih.gov/protein/SQW37114.1?report=genbank&log$=protalign&blast_rank=1&RID=YKTNX4XS014) | Turkey feces |
| VRES0584 | [SQX38949.1](https://www.ncbi.nlm.nih.gov/protein/SQX38949.1?report=genbank&log$=protalign&blast_rank=1&RID=YKTNX4XS014) | Turkey feces |
| VRES0585 | [SQY07345.1](https://www.ncbi.nlm.nih.gov/protein/SQY07345.1?report=genbank&log$=protalign&blast_rank=1&RID=YKTNX4XS014) | Turkey feces |
| VRES0533 | [SRY84987.1](https://www.ncbi.nlm.nih.gov/protein/SRY84987.1?report=genbank&log$=protalign&blast_rank=1&RID=YKTNX4XS014) | Turkey feces |
| VRES0536 | [SQW01088.1](https://www.ncbi.nlm.nih.gov/protein/SQW01088.1?report=genbank&log$=protalign&blast_rank=1&RID=YKTNX4XS014) | Turkey feces |
| VRES0580 | [SQW89426.1](https://www.ncbi.nlm.nih.gov/protein/SQW89426.1?report=genbank&log$=protalign&blast_rank=1&RID=YKTNX4XS014) | Turkey feces |
| VRES0535 | [SQW64783.1](https://www.ncbi.nlm.nih.gov/protein/SQW64783.1?report=genbank&log$=protalign&blast_rank=1&RID=YKTNX4XS014) | Turkey feces |
| VRES0668 | [SQZ94674.1](https://www.ncbi.nlm.nih.gov/protein/SQZ94674.1?report=genbank&log$=protalign&blast_rank=1&RID=YKTNX4XS014) | Turkey feces |
| VRES0568 | [SQX57971.1](https://www.ncbi.nlm.nih.gov/protein/SQX57971.1?report=genbank&log$=protalign&blast_rank=1&RID=YKTNX4XS014) | Turkey feces |
| VRES0552 | [SRA59934.1](https://www.ncbi.nlm.nih.gov/protein/SRA59934.1?report=genbank&log$=protalign&blast_rank=1&RID=YKTNX4XS014) | Turkey feces |
| VRES0570 | [SQX27250.1](https://www.ncbi.nlm.nih.gov/protein/SQX27250.1?report=genbank&log$=protalign&blast_rank=1&RID=YKTNX4XS014) | Turkey feces |
| VRES0573 | [SQY17530.1](https://www.ncbi.nlm.nih.gov/protein/SQY17530.1?report=genbank&log$=protalign&blast_rank=1&RID=YKTNX4XS014) | Turkey feces |
| VRES0581 | [SQV73047.1](https://www.ncbi.nlm.nih.gov/protein/SQV73047.1?report=genbank&log$=protalign&blast_rank=1&RID=YKTNX4XS014) | Turkey feces |
| VRES0559 | [SQY77711.1](https://www.ncbi.nlm.nih.gov/protein/SQY77711.1?report=genbank&log$=protalign&blast_rank=1&RID=YKTNX4XS014) | Turkey feces |
| VRES0572 | [SQY89347.1](https://www.ncbi.nlm.nih.gov/protein/SQY89347.1?report=genbank&log$=protalign&blast_rank=1&RID=YKTNX4XS014) | Turkey feces |
| VRES0532 | [SQW23350.1](https://www.ncbi.nlm.nih.gov/protein/SQW23350.1?report=genbank&log$=protalign&blast_rank=1&RID=YKTNX4XS014) | Turkey feces |
| SC371 | [RDQ09216.1](https://www.ncbi.nlm.nih.gov/protein/RDQ09216.1?report=genbank&log$=protalign&blast_rank=1&RID=YKTNX4XS014) | Surface water lake Superior |
| VRES0562 | [SVF57037.1](https://www.ncbi.nlm.nih.gov/protein/SVF57037.1?report=genbank&log$=protalign&blast_rank=1&RID=YKTNX4XS014) | Turkey feces |
| VRES0586 | [SVF61232.1](https://www.ncbi.nlm.nih.gov/protein/SVF61232.1?report=genbank&log$=protalign&blast_rank=1&RID=YKTNX4XS014) | Turkey feces |

**Supplemental Table 2.**

**List of *E. coli* strains that contain Tag genomic islands - (*tagB* and *tagC* genes)**

| **Strain/isolate** | **Accession number** | **Information or source** |
| --- | --- | --- |
| DA33135 | CP029576.1 | Clinical source, Sweden |
| Ecol_448 | CP015076.1 | Clinical source, Argentina |
| Ecol_743 | CP015069.1 | Human isolate, Dubai |
| SE15 | AP009378.1 | Human commensal, B2 group [1] |
| Ecol_745 | CP015074.2 | Human isolate, Morocco |
| Eco 889 | CP015159.1 | NIH, human urine |
| MVAST0167 | CP014492.1 | ST131, Minnesota |
| 55989 | CP028304.1 | UTI, Pakistan |
| AR_451 | CP030337.1 | Antimicrobial resistant strain |
| E41-1 | CP028483.1 | Human sputum, Shanghai |
| AR_0081 | CP027534.1 | Antimicrobial resistant |
| NQ3 | CP024720.1 | Domestic yak |
| LS4 | CP024717.1 | Domestic yak |
| AR_0055 | CP021935.1 | Antimicrobial resistant |
| \| H105 \|  \| \| --- \| --- \| | CP021454.1 | ST131, vaginal swab  Germany |
| AR_0058 | CP021689.1 | Antimicrobial resistant |
| 81009 | CP021179.1 | O25b:H4 urine isolate  United Arab Emitrates |
| AR_0104 | CP020116.1 | Antimicrobial resistant |
| Ecol_AZ162 | CP019015.1 | Antimicrobial resistant, human, Boston |
| Ecol_867 | CP019000.1 | Antimicrobial resistant, Toronto, Canada |
| Ecol_656 | CP018979.1 | Human Beijing, China |
| Ecol_542 | CP018970.1 | Vietnam |
| Ecol_276 | CP018953.1 | Human Antimicrobial resistant, U.S.A. |
| NCTC 13441 | LT632320.1 | O25b:H4 ST131 uropathogenic strain, CTX-M-15 |
| O25b:H4 | CP015085.1 | Urinary tract pathogenic, Saudi Arabia |
| Ecol 732 | CP015138.1 | Human AR strain, Bangkok |
| JJ1887 | CP014316.1 | UTI, ST131 strain [2] |
| ZH193 | CP014497.1 | Human ST131, New York |
| JJ2434 | CP013835.1 | Human Minneapolis |
| CD306 | CP013831.1 | ST131 from a Cat, New York |
| [P46212](https://blast.ncbi.nlm.nih.gov/Blast.cgi#alnHdr_966626806) | CP013658.1 | Oxford, U.K. human urine [3] |
| EC958 | HG941718.1 | UTI, blood, England [4] |
| JJ1886 | CP006784.1 | USA, fatal sepsis [5] |
| Z247 | CP021207.1 | Human blood, China [6] |
| Ecol 244 | CP019020.1 | Argentina, human clinical |
| G749 | CP014488.1 | Human clinical, Seattle Washington |
| MNCRE44 | CP010876.1 | Human ST131 sputum [7] |
| ZH063 | CP014522.1 | Human ST131, Winnipeg, Canada |
| Ecol AZ159 | CP019008.1 | Human Bogota, Columbia  Human ST131, Minneapolis |
| SaT040 | CP014495.1 | Human ST131, Burlingtonm, VT |
| JJ1897 | CP013837.1 | Human ST131, Minneapolis |
| PCN033 | CP014488.1 | O11 group D, PorcineExPEC [8] |
| FHI40 | LM996283.1 | Non-O157 STEC human Norway |
| RM9387 | CP009104.1 | O104 STEC, cattle feces [9] |
| M18 | CP010219.1 | Mouse feces, China |
| CI5 | CP011018.1 | Uropathogenic strain, pyelonephritis [10] |
| M3 | CP010183.1 | Mouse feces, China |
| M1 | CP010180.1 | Mouse feces, China |
| M8 | [CP010191.1](https://www.ncbi.nlm.nih.gov/nucleotide/CP010191.1?report=genbank&log$=nuclalign&blast_rank=51&RID=S2WT0K20014) | Mouse feces, China |

**References**

1. Toh, H., et al., *Complete genome sequence of the wild-type commensal Escherichia coli strain SE15, belonging to phylogenetic group B2.* Journal of bacteriology, 2010. **192**(4): p. 1165-1166.

2. Johnson, T.J., et al., *Complete genome sequence of a CTX-M-15-producing Escherichia coli strain from the H30Rx subclone of sequence type 131 from a patient with recurrent urinary tract infections, closely related to a lethal urosepsis isolate from the patient's sister.* Genome announcements, 2016. **4**(3): p. e00334-16.

3. Stoesser, N., et al., *Evolutionary history of the global emergence of the Escherichia coli epidemic clone ST131.* MBio, 2016. **7**(2): p. e02162-15.

4. Forde, B.M., et al., *The complete genome sequence of Escherichia coli EC958: a high quality reference sequence for the globally disseminated multidrug resistant E. coli O25b: H4-ST131 clone.* PLoS One, 2014. **9**(8): p. e104400.

5. Andersen, P.S., et al., *Complete genome sequence of the epidemic and highly virulent CTX-M-15-producing H30-Rx subclone of Escherichia coli ST131.* Genome announcements, 2013. **1**(6): p. e00988-13.

6. Zheng, B., et al., *Coexistence of MCR-1 and NDM-1 in clinical Escherichia coli isolates.* Clinical Infectious Diseases, 2016. **63**(10): p. 1393-1395.

7. Johnson, T.J., et al., *Complete genome sequence of a carbapenem-resistant extraintestinal pathogenic Escherichia coli strain belonging to the sequence type 131 H30R subclade.* Genome announcements, 2015. **3**(2): p. e00272-15.

8. Liu, C., et al., *Genome analysis and in vivo virulence of porcine extraintestinal pathogenic Escherichia coli strain PCN033.* BMC genomics, 2015. **16**(1): p. 717.

9. Yan, X., et al., *Genome sequencing and comparative genomics provides insights on the evolutionary dynamics and pathogenic potential of different H-serotypes of Shiga toxin-producing Escherichia coli O104.* BMC microbiology, 2015. **15**(1): p. 83.

10. Mehershahi, K.S., S.N. Abraham, and S.L. Chen, *Complete genome sequence of uropathogenic Escherichia coli strain CI5.* Genome announcements, 2015. **3**(3): p. e00558-15.
