# Supplementary figures and images for: Three new serine-protease autotransporters of Enterobacteriaceae (SPATEs) from extra-intestinal pathogenic Escherichia coli and combined role of SPATEs for cytotoxicity and colonization of the mouse kidney
Source: Virulence. 2019 Jun 14;10(1):568–87. doi: 10.1080/21505594.2019.1624102 (PMC6592367; doi:10.1080/21505594.2019.1624102)

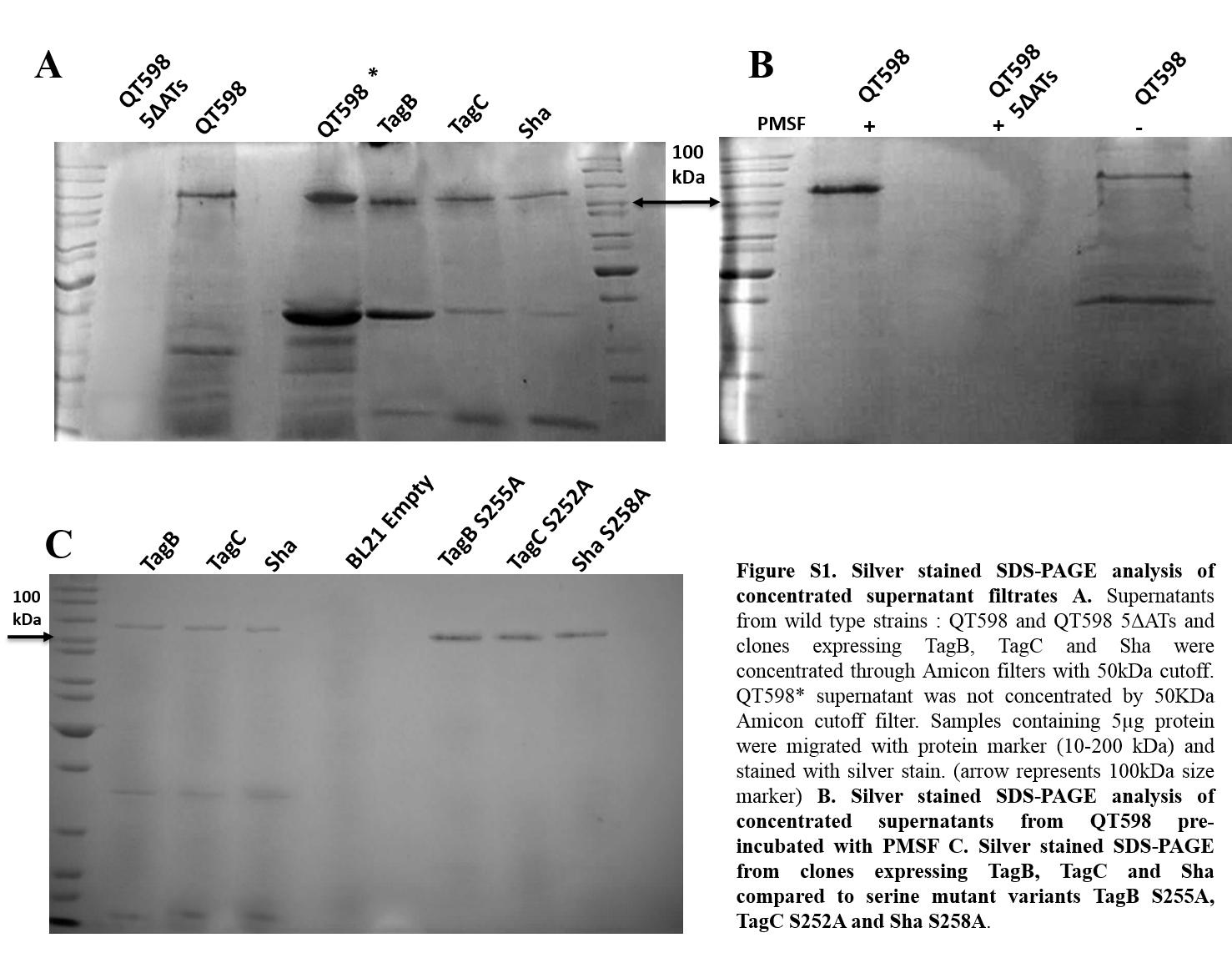

Supplement: Supplemental Material [file kvir-10-01-1624102-s001.zip › Figure_S1.tif]

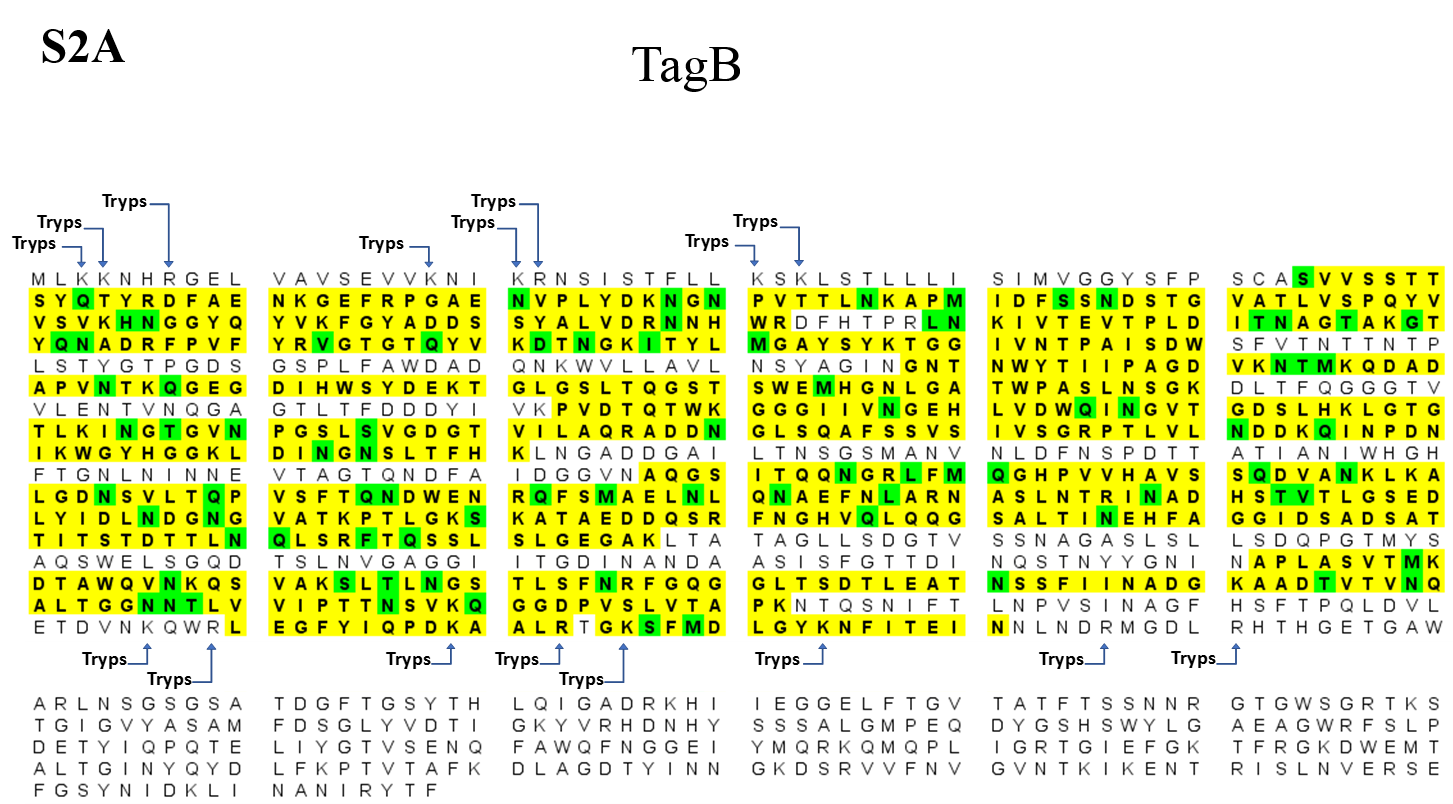

Supplement: Supplemental Material [file kvir-10-01-1624102-s001.zip › Figure_S2A.tif]

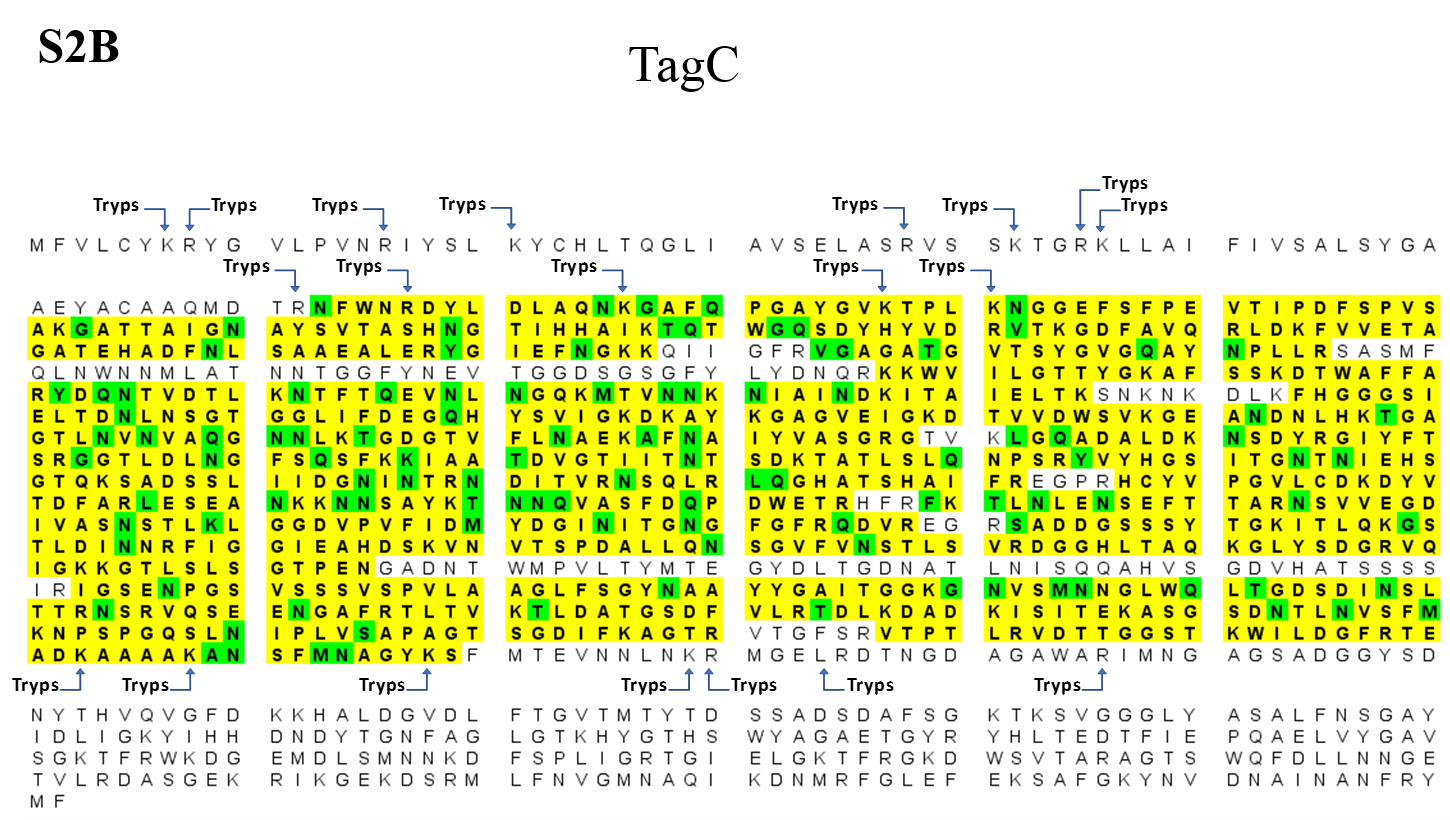

Supplement: Supplemental Material [file kvir-10-01-1624102-s001.zip › Figure_S2B.tif]

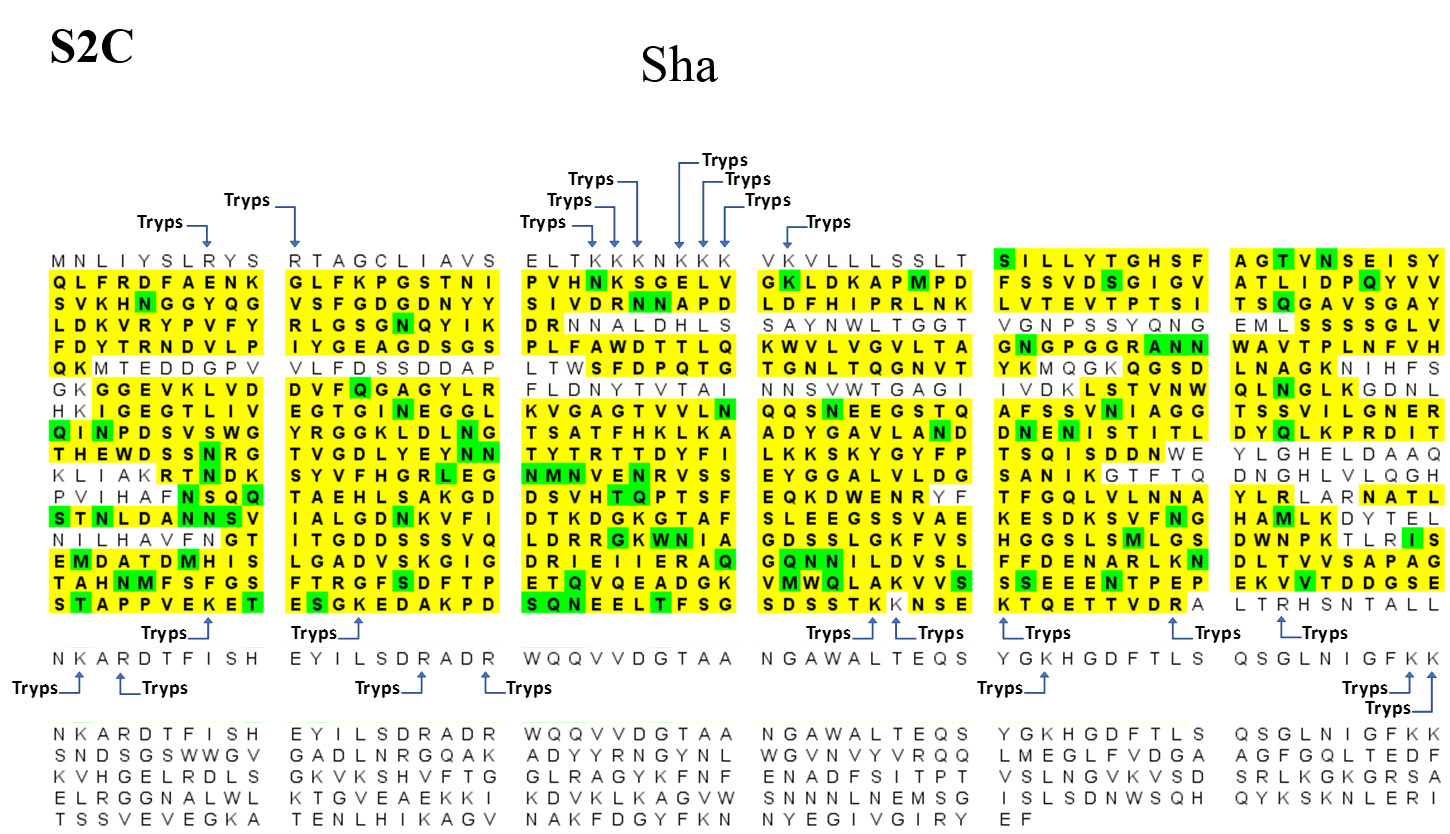

Supplement: Supplemental Material [file kvir-10-01-1624102-s001.zip › Figure_S2C.tif]

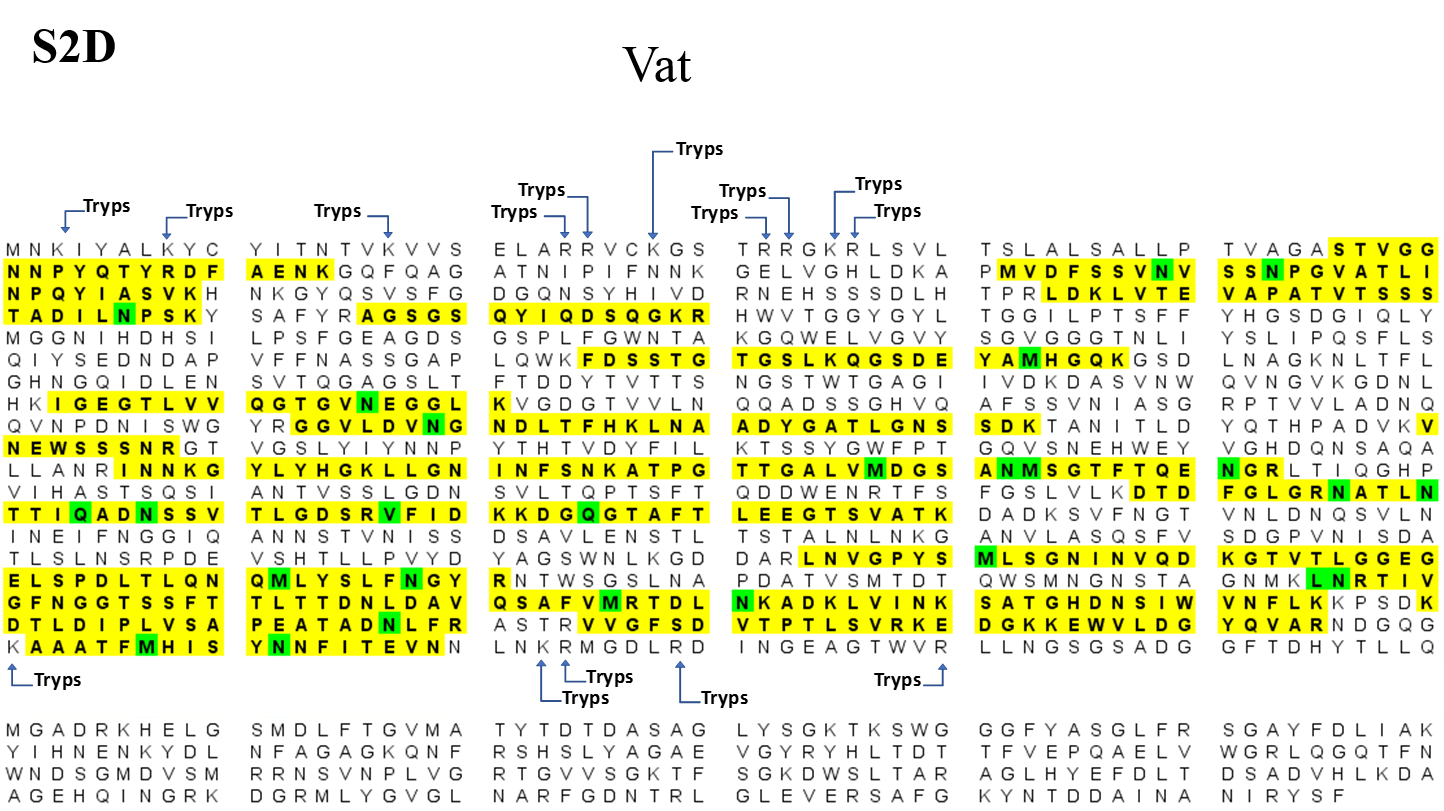

Supplement: Supplemental Material [file kvir-10-01-1624102-s001.zip › Figure_S2D.tif]

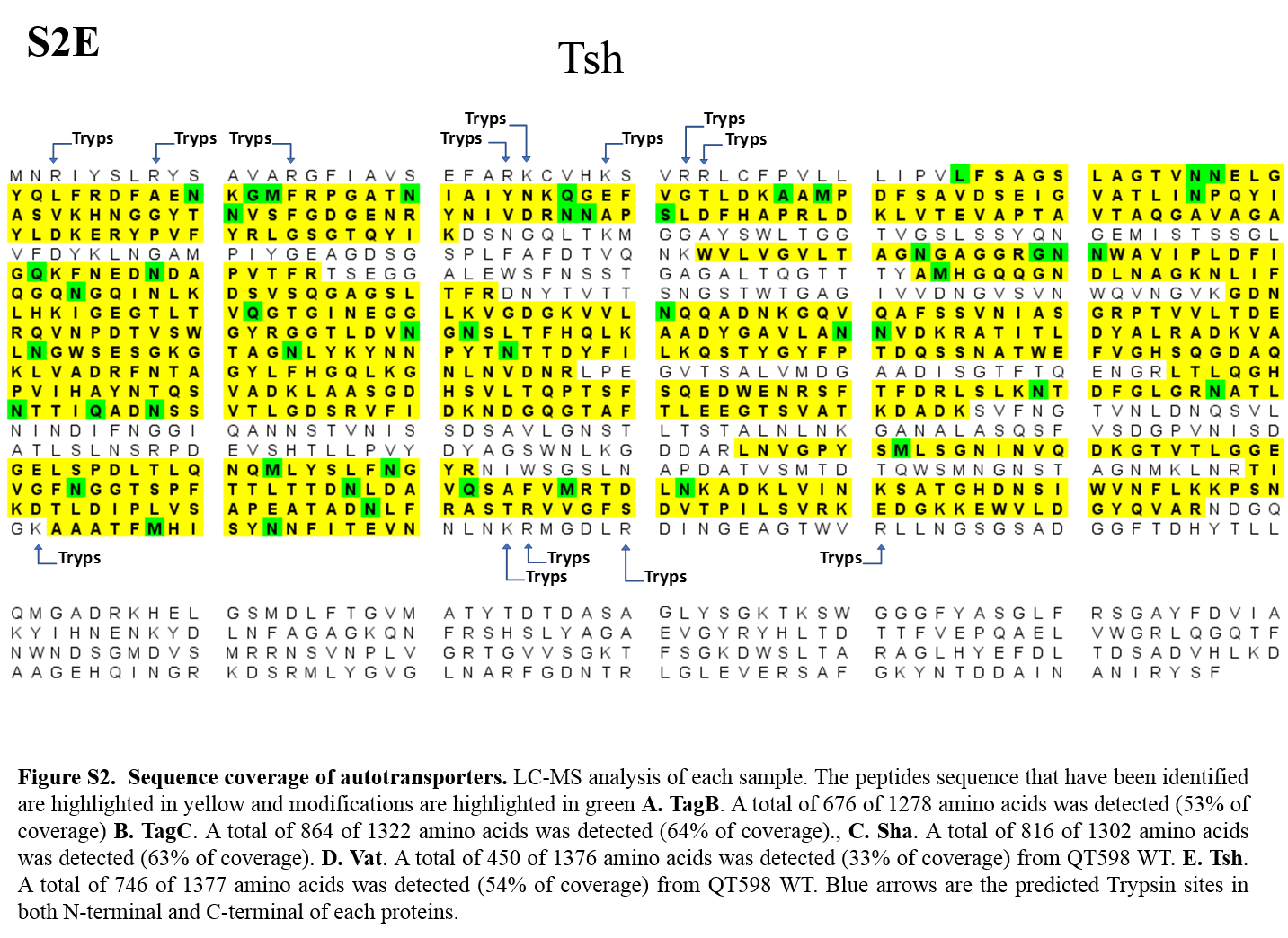

Supplement: Supplemental Material [file kvir-10-01-1624102-s001.zip › Figure_S2E.tif]

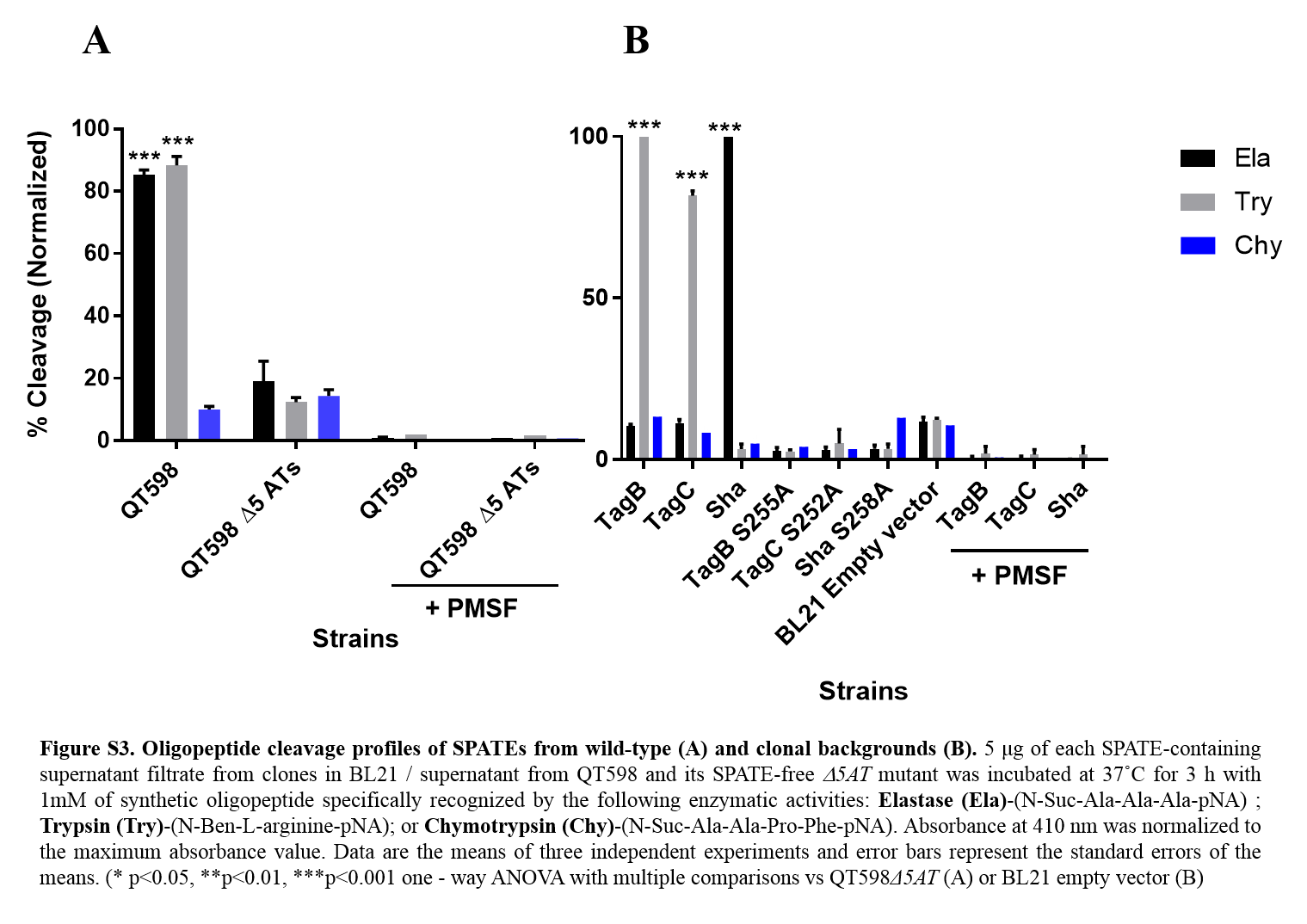

Supplement: Supplemental Material [file kvir-10-01-1624102-s001.zip › Figure_S3.tif]

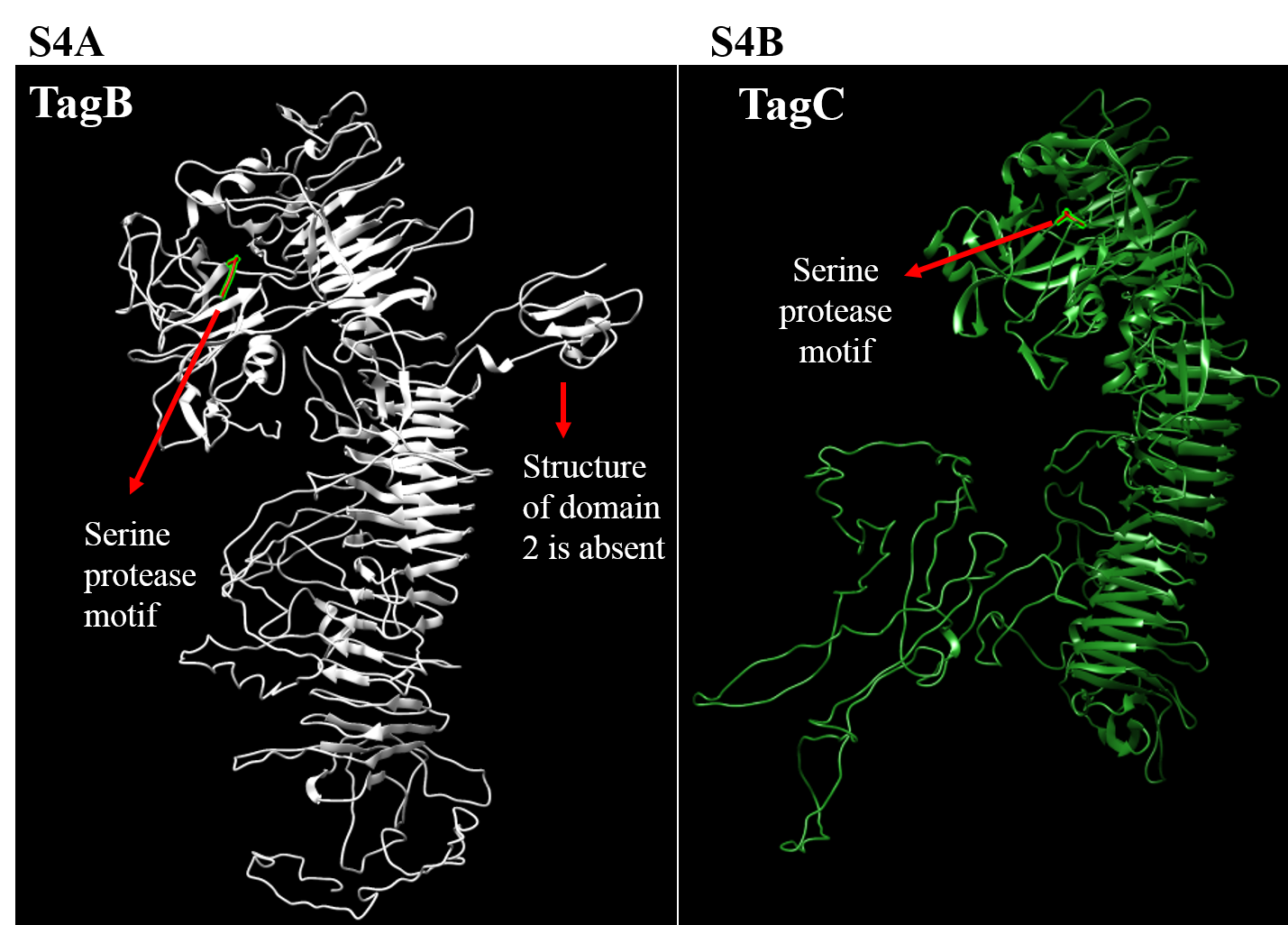

Supplement: Supplemental Material [file kvir-10-01-1624102-s001.zip › Figure_S4A_S4B.tif]

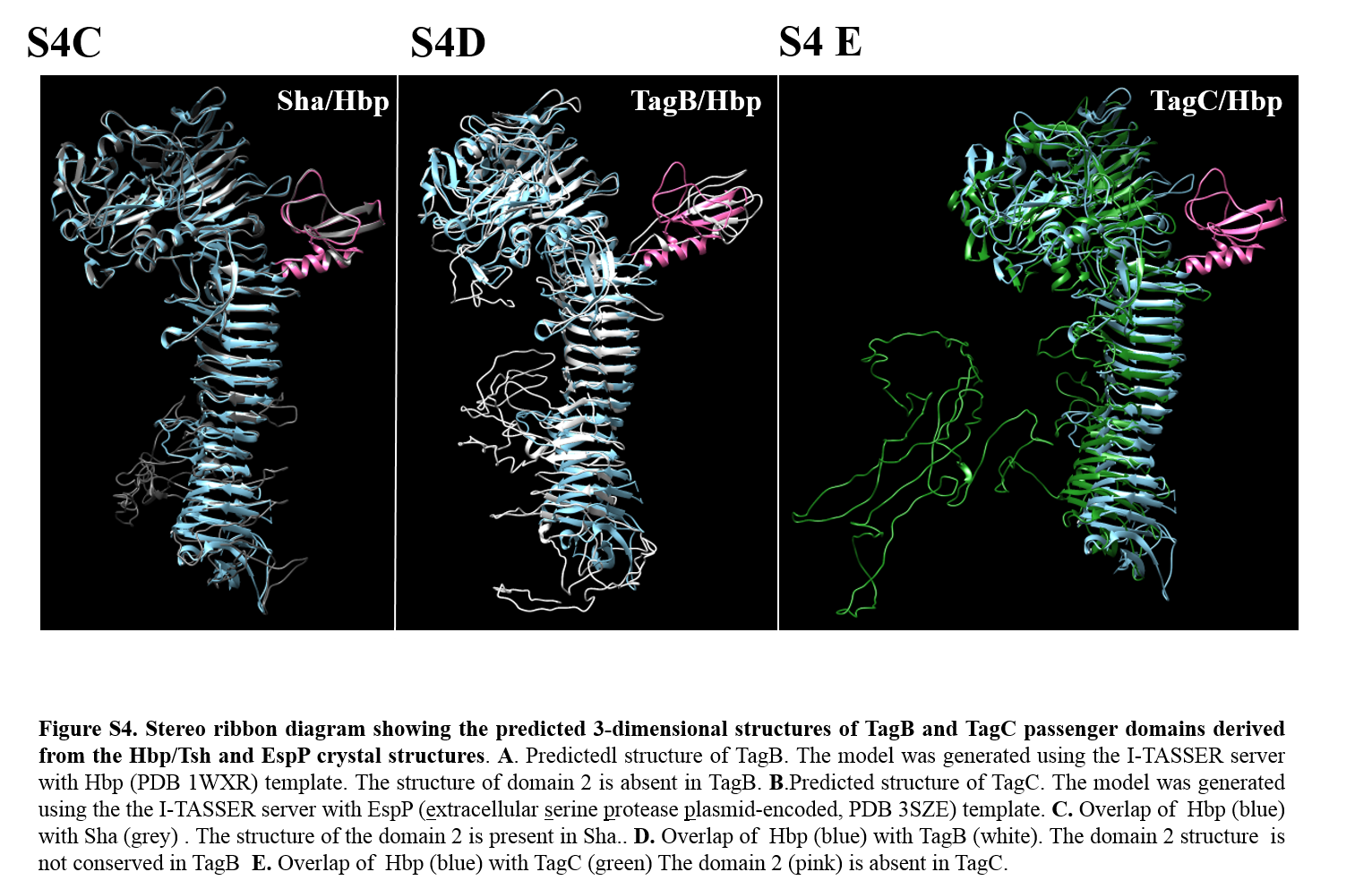

Supplement: Supplemental Material [file kvir-10-01-1624102-s001.zip › Figure_S4C_S4D_S4E.tif]

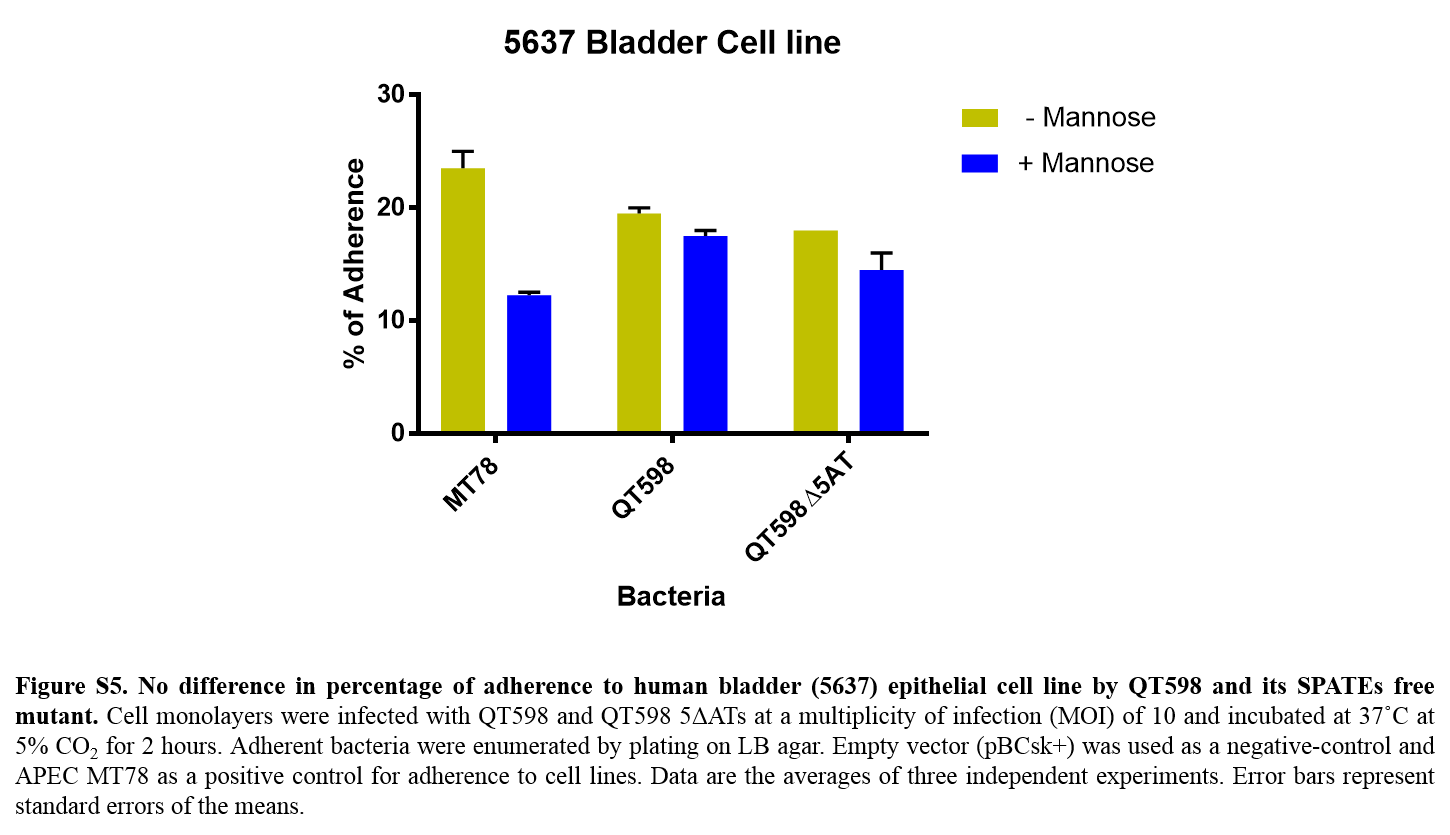

Supplement: Supplemental Material [file kvir-10-01-1624102-s001.zip › Figure_S5.tif]
